# Characterization of novel bacteriophages for effective phage therapy against *Vibrio* infections in aquaculture

**DOI:** 10.71150/jm.2502009

**Published:** 2025-05-27

**Authors:** Kira Moon, Sangdon Ryu, Seung Hui Song, Se Won Chun, Nakyeong Lee, Aslan Hwanhwi Lee

**Affiliations:** Division of Environmental Materials, Honam National Institute of Biological Resources, Mokpo 58762, Republic of Korea

**Keywords:** bacteriophage, phage therapy, *Vibrio* spp., aquaculture, antibiotic replacement

## Abstract

The widespread use of antibiotics in aquaculture has led to the emergence of multidrug-resistant pathogens and environmental concerns, highlighting the need for sustainable, eco-friendly alternatives. In this study, we isolated and characterized three novel bacteriophages from aquaculture effluents in Korean shrimp farms that target the key *Vibrio* pathogens, *Vibrio harveyi*, and *Vibrio parahaemolyticus*. Bacteriophages were isolated through environmental enrichment and serial purification using double-layer agar assays. Transmission electron microscopy revealed that the phages infecting *V. harveyi*, designated as vB_VhaS-MS01 and vB_VhaS-MS03, exhibited typical *Siphoviridae* morphology with long contractile tails and icosahedral heads, whereas the phage isolated from *V. parahaemolyticus* (vB_VpaP-MS02) displayed *Podoviridae* characteristics with an icosahedral head and short tail.

Whole-genome sequencing produced complete, circularized genomes of 81,710 bp for vB_VhaS-MS01, 81,874 bp for vB_VhaS-MS03, and 76,865 bp for vB_VpaP-MS02, each showing a modular genome organization typical of Caudoviricetes. Genomic and phylogenetic analyses based on the terminase large subunit gene revealed that although vB_VhaS-MS01 and vB_VhaS-MS03 were closely related, vB_VpaP-MS02 exhibited a distinct genomic architecture that reflects its unique morphology and host specificity. Collectively, these comparative analyses demonstrated that all three phages possess genetic sequences markedly different from those of previously reported bacteriophages, thereby establishing their novelty. One-step growth and multiplicity of infection (MOI) experiments demonstrated significant differences in replication kinetics, such as burst size and lytic efficiency, among the phages, with vB_VhaS-MS03 maintaining the most effective bacterial control, even at an MOI of 0.01. Additionally, host range assays showed that vB_VhaS-MS03 possessed a broader spectrum of activity, supporting its potential use as a stand-alone agent or key component of phage cocktails. These findings highlight the potential of region-specific phage therapy as a targeted and sustainable alternative to antibiotics for controlling *Vibrio* infections in aquaculture.

## Introduction

Bacterial strains belonging to the family *Vibrionaceae* within the class Gammaproteobacteria are among the most common pathogens found in marine environments. The well-known genus *Vibrio* comprises a group of waterborne pathogens that can trigger outbreaks, particularly among fish in aquaculture facilities (Thompson et al., 2004). Among the *Vibrio* species, *V. parahaemolyticus* and *V. harveyi* are the most frequently detected pathogens in aquaculture systems, whereas other *Vibrio* species, such as *V. vulnificus* and *V. alginolyticus*, can also cause infections but are typically associated with different ecological niches or have a lower prevalence in aquaculture environments (Sheikh et al., 2024; Zhang et al., 2020). These outbreaks not only result in substantial economic losses due to mass mortalities of farmed species but also pose significant challenges for disease management. A notable example of the impact of *Vibrio*-induced diseases occurred between May and August 2024, when an outbreak of acute hepatopancreatic necrosis disease (AHPND) was reported in whiteleg shrimp farms in South Korea (Director of the National Fisheries Quality Management Service, 2024). In response, the South Korean National Fisheries Quality Management Service of the Ministry of Oceans and Fisheries issued an official disease outbreak warning. AHPND is caused by the toxins, PirA and PirB, which are encoded by virulence genes in *V. parahaemolyticus* (Kumar et al., 2021) and infect the hepatopancreas and digestive tract of shrimp, leading to tissue necrosis and potentially causing up to 100% mortality. This outbreak highlights the urgent need for effective management strategies to mitigate the economic losses associated with *Vibrio*-induced diseases.

Such outbreaks often result in mass mortality of aquaculture species and significant financial losses for fish farmers. Although infections originating from aquaculture may be transmitted to humans through consumption and generally manifest with mild or no symptoms (Sanches-Fernandes et al., 2022), severe cases—characterized by gastroenteritis, wound or ear infections, and septicemia—can occur (Letchumanan et al., 2016). These symptoms are generally treatable with antibiotics, making vibriosis manageable in both humans and aquaculture species. However, the widespread use of antibiotics in aquaculture as a prophylactic or pre-treatment measure has not only reduced the risk of bacterial diseases, such as those caused by *Vibrio* spp., but has also contributed to the emergence of antibiotic-resistant pathogens. For instance, Liu et al. (2024) reported that continuous antibiotic application to shrimp cultures led to increased antibiotic resistance in *V. harveyi*. Consequently, the deployment of second- and third-line antibiotics has become necessary, inadvertently leading to a rise in multidrug-resistant strains and an increasing number of affected patients. Moreover, excessive antibiotic use results in the accumulation of residual antibiotics in the environment and in fish, ultimately leading to human exposure through consumption (Cabello, 2006). These residual antibiotics further drive the proliferation and dissemination of antibiotic-resistance genes within bacterial populations. Therefore, safe and environmentally friendly alternatives to antibiotics are urgently needed.

Bacteriophages (phages), viruses that infect bacteria, have long been recognized for their potential in phage therapy (Sulakvelidze et al., 2001). Interestingly, as bacteria acquire antibiotic resistance, they often become more susceptible to phage infections, rendering phage therapy particularly effective against antibiotic-resistant pathogens (Hill et al., 2018). Despite its potential, the industrial application of phage therapy has been hindered by concerns over delayed efficacy when compared to antibiotics and the higher associated costs (Gordillo Altamirano and Barr, 2019). Nevertheless, persistent environmental contamination by residual antibiotics and the escalating emergence of multidrug-resistant pathogens have renewed interest in phage therapy as a viable alternative. Phages can infect specific bacterial hosts without affecting other members of the microbial community (Stone et al., 2019). Consequently, phage therapy has been recognized for its ability to selectively eliminate targeted bacterial pathogens while preserving beneficial probiotics that support fish growth (Kowalska et al., 2020). Moreover, because phages exclusively infect bacterial cells and do not harm eukaryotic cells, phage therapy is considered a safe, consumable alternative to antibiotics. However, as phage therapy removes pathogens much slower than antibiotics do, its implementation in the aquaculture industry has been hindered. Recent advancements in phage therapy have been implemented not only in clinical settings, such as in the treatment of lung infections and burn wounds, but also in the agricultural and aquaculture sectors (Culot et al., 2019). For example, phages targeting *Erwinia amylovora*, the causative agent of fire blight in *Rosaceae* plants that affects apple and pear production (Piqué et al., 2015), have been isolated and developed as eco-friendly alternatives to traditional antibiotics (Park et al., 2022).

In light of these challenges and opportunities, our study focused on isolating and characterizing novel bacteriophages from aquaculture effluents in Korean shrimp farms. These phages target *V. parahaemolyticus* and *V. harveyi*, which are pathogens responsible for devastating outbreaks, such as AHPND. In addition, because bacteria and phages have coevolved in the natural environment and engage in a continuous arms race, phages are uniquely adapted to track the evolutionary changes of their bacterial hosts (Koskella and Brockhurst, 2014). As pathogenic bacteria acquire antibiotic resistance, phages concurrently evolve, highlighting the importance of identifying naturally occurring phages that target specific pathogens in aquaculture systems. Furthermore, although bacterial strains often exhibit high genetic similarity, regional ecotypes can differ, making local bacterial populations distinct (Meyer et al., 2016). Therefore, isolating phages from the Korean aquaculture environment is likely to yield candidates that are well-suited and specific for controlling vibriosis-causing strains in South Korea. By developing a tailored phage therapy system, we aimed to provide an effective and environmentally friendly alternative to conventional antibiotic treatments. The primary objectives of this study were to isolate novel phages from aquaculture systems in Korean shrimp farms and evaluate their lytic activity against *Vibrio* spp. This research not only contributes to the growing body of evidence supporting phage therapy, but also addresses the critical need for sustainable disease management in aquaculture.

## Materials and Methods

### Host bacterial strain culture and phage isolation

The *V. harveyi* (KCTC12724) and *V. parahaemolyticus* (KCTC2729) strains used as hosts in this study were obtained from the Korean Collection for Type Cultures (KCTC, Korea). The strains were cultured on marine agar (MA; BD, USA) at 25°C. Prior to phage cultivation, the hosts were grown in marine broth (MB; BD) until reaching an optical density at 600 nm (OD_600_) of approximately 0.2 in a shaking incubator. To isolate the novel *Vibrio* phages, 10 L surface water was collected in July 2021 from a shrimp aquaculture plant in Jindo Province, Korea (34.537306°N, 126.344365°E). Shrimp breeding water and effluent samples were collected in sterile bottles and maintained at 4°C during transport to the laboratory. Filtration and subsequent analyses were performed immediately upon arrival at the laboratory. The environmental conditions at the time of sample collection were recorded as a temperature of 17°C, pH 7.6, and conductivity of 320 μS/cm (YSI ProDSS Multiparameter Digital Water Quality Meter; YSI Incorporated, USA). The samples were initially filtered through a 0.2-μm pore-size filter (SUPOR; Millipore, USA) to remove bacterial cells and particulate matter. The resulting filtrates were used for both the enrichment and double-layer agar (DLA) assays to screen for and isolate *Vibrio* phages. The DLA assay enables direct plaque formation, whereas enrichment enhances the detection of low-abundance phages.

### Phage enrichment and purification

Phage particles in the 0.2-μm filtrate were enriched prior to screening (Nair et al., 2022). Briefly, 20 ml of 5×-concentrated MB was added to 80 ml of the filtrate, followed by addition of 10 ml liquid host culture (OD_600_ ≈ 0.2). This mixture was incubated at 25°C for two weeks to allow phages to infect the host bacteria. During incubation, 5 ml aliquots were collected every five days; to each aliquot, 3 ml chloroform was added to lyse and remove the bacterial cells. After vortexing for 1 min, the samples were centrifuged at 3,000 × g for 30 min to pellet the bacterial debris. The clear supernatant was carefully transferred to a clean tube and stored at 4°C.

Phage screening was performed using a spot-on DLA assay (Song et al., 2021). The bottom layer was prepared with 1.5× MA in a sterile Petri dish. For the top layer, 9 ml molten 0.7× MA was mixed with 1 ml liquid host culture (OD_600_ ≈ 0.2) and gently poured over the solidified bottom layer. After the top agar layer had solidified, 10 μl of the chloroform-treated enriched sample was spotted onto the surface and allowed to absorb. The plates were then incubated at 25°C for 24 h; the appearance of plaques indicated the presence of phage particles.

Individual plaques were picked using sterile tips and suspended in SM buffer (100 mM NaCl, 8 mM MgSO₄∙7H₂O, 50 mM Tris-Cl, 0.01% [w/v] gelatin, pH 7.5) containing 20 μl chloroform to eliminate residual bacterial cells. For phage purification, serial DLA assays were performed at least thrice. In each round, a thin layer of 1.5× MA was poured into a Petri dish and allowed to solidify. Then, 9 ml molten 0.7× MA was mixed with 1 ml of the liquid host culture (OD₆₀₀ ≈ 0.2) and 100 μl of the phage suspension, and the mixture poured over the bottom layer. Following incubation at 25°C for 24 h, well-isolated plaques were picked, as previously described. This serial purification yielded single-genotype phage samples, which were stored in SM buffer at 4°C until further analysis. For long-term storage, phage suspensions in SM buffer (with 20 μl chloroform) were mixed with an equal volume of sterilized 50% glycerol and stored at -80°C.

### Phage amplification for transmission electron microscopy observation and DNA extraction

For genetic and morphological analyses, phage concentrates were prepared from a 500-ml liquid culture at the lysate stage when the host bacterial concentration reached approximately half of that in the stationary phase. To the culture, 10% (w/v) polyethylene glycol (PEG) 8000 and 1 M NaCl were added (Moon et al., 2017). The mixture was gently agitated, incubated on ice overnight, and then centrifuged at 11,000 × g for 40 min. An equal volume of chloroform was added, and the mixture vortexed and centrifuged at 3,000 rpm for 30 min at 4°C to remove residual PEG. The supernatant was recovered and ultracentrifuged at 250,000 × g in a Beckman Coulter Optima AUC ultracentrifuge (SW 55 Ti rotor; Beckman Coulter, USA). The resulting virus pellet was resuspended in 200 μl SM buffer, carefully recollected, and then stored at 4°C.

For transmission electron microscopy (TEM), the phage concentrates were adsorbed onto formvar- and carbon-coated copper grids (Electron Microscopy Sciences, USA) for 3 min, then negatively stained with 2% uranyl acetate. The samples were examined using a Hitachi H-7650 transmission electron microscope at the Center for University-Wide Research Facilities (Jeonbuk National University, Korea). Morphological taxonomic classification was performed based on particle morphology and size, according to the guidelines of the International Committee on Taxonomy of Viruses (Gorbalenya et al., 2020).

Genomic DNA was extracted from the phage concentrates using a Qiagen DNeasy Blood and Tissue Kit (Qiagen, Germany), with minor modifications. Specifically, 70 μl phage concentrate was mixed with 30 μl proteinase K, 3 μl RNase A, and 300 μl ATL buffer, then incubated at 56°C to degrade viral capsid proteins. Next, 300 μl AL buffer and 100% ethanol were added, and the mixture processed through a purification column for washing and DNA elution. The sequencing library was prepared using a TruSeq DNA sample preparation kit and sequenced on an Illumina HiSeq platform with 150-bp paired-end reads by Macrogen (Korea).

### Genome and phylogenetic analysis of the phages

Genomic DNA from each of the three novel phages isolated was sequenced on an Illumina HiSeq platform, yielding approximately 1 Gb paired-end reads per phage. Raw reads were processed using Trimmomatic v.0.39 (Bolger et al., 2014) to remove adapter sequences and low-quality regions, with a minimum read length of 150 bp. The quality-filtered reads were assembled de novo using SPAdes v3.15.5 (spades.py) (Bankevich et al., 2012), generating an initial set of contigs that were manually curated and scaffolded into a single, closed genome per phage using Bandage v0.8.1 (Wick et al., 2015). Open reading frames (ORFs) were predicted using Prokka v1.14.6 (Seemann, 2014), and functional annotation subsequently performed via BLASTp searches against the National Center for Biotechnology Information (NCBI) nonredundant (nr) protein database. Circular genome maps detailing the GC content, gene distribution, and ORF annotations were generated using the GCView tool (Grin and Linke, 2011). To assess genomic relatedness between the newly isolated *Vibrio* phages and other reference phage genomes, a pairwise average nucleotide identity (ANI) was computed. Briefly, the complete genome sequences of each phage were compared with those of closely related *Vibrio* phages obtained from the NCBI database. ANI was calculated using the ANI Calculator (Yoon et al., 2017), with default parameters.

For phylogenetic analysis, the terminase large subunit gene (*terL*) was extracted from each genome and aligned using MUSCLE 5 (Edgar, 2022). A maximum-likelihood phylogenetic tree was constructed using IQ-TREE v2.0.6 (Nguyen et al., 2015), and the robustness of the inferred topology evaluated with 1,000 bootstrap replicates. Finally, the complete genome sequences were deposited in the NCBI database under the following accession numbers: vB_VhaS-MS01 (OR102880), vB_VpaP-MS02 (OR102882), and vB_VhaS-MS03 (OR102881).

### One-step growth assay and multiplicity of infection assay of vibriophages

To determine the life cycle parameters and burst size of the phages, a one-step growth assay was performed (Kropinski, 2018). Host bacteria were cultured in liquid medium until they reached an OD_600_ of 0.2. Then, 1 ml aliquots of the bacterial culture were mixed with phage stock at a multiplicity of infection (MOI) of approximately 0.1 (with bacterial and phage concentrations of ~4~6 × 10^9^ cells/ml and ~4~6 × 10^8^ particles/ml, respectively) and incubated at 25°C for 10 min. The mixture was centrifuged at 3,000 rpm for 10 min, the supernatant discarded, and the pellet then resuspended in 40 ml MB. Immediately after resuspension, a 1 ml sample was collected for the plaque assay. Samples were collected every 5 min for the first 30 min and every 10 min thereafter until 90 min. Each subsample was analyzed using the DLA plaque assay, with plates incubated at 25°C overnight. Plaque counts were used to calculate burst size, and each assay performed in triplicate for each vibriophage. The burst size (*B*) of each phage was determined using the following equation:


$$B\;=\;\frac{{\text{PFU}}_{\text{plateau}}}{{\text{PFU}}_{t=0}}$$

To identify the lowest MOI that results in effective host bacterial lysis, co-culture experiments were conducted at MOIs of 10, 1, 0.1, and 0.01 (Necel et al., 2021). For each MOI, 1 ml bacterial culture (OD₆₀₀ ≈ 0.2) was mixed with phages at the corresponding concentrations and added to 10 ml MB. After gentle mixing, 200 μl aliquots were transferred into a 96-well plate, and bacterial growth monitored by measuring OD₆₀₀ values using a BioTek Synergy H1 Multimode Reader (Agilent Technologies, USA) at 25°C. For vB_VhaS-MS01 and vB_VpaP-MS02, readings were recorded every 30 min for 480 min, whereas for vB_VhaS-MS03, measurements were taken hourly.

### Determination of the host range of susceptible bacteria

Phages vB_VhaS-MS01, vB_VpaP-MS02, and vB_VhaS-MS03 were evaluated for their ability to infect other bacterial hosts in addition to their original hosts. Therefore, the major pathogens responsible for vibriosis in aquaculture, *V. alginolyticus* (KCTC2472), *V. anguillarum* (KCTC2711), and *V. vulnificus* (KACC15317), were selected for testing and acquired from the KCTC and Korean Agricultural Culture Collection (KACC, Korea). The *V. harveyi* and *V. parahaemolyticus* strains were also tested for potential cross-infectivity by the phages. Each bacterial strain was incubated in MA at 25 or 37°C, according to the characteristics of each host, until they reached an OD_600_ value of 0.2. Then, each phage was added to the bacterial culture at an MOI of 1 and placed in a shaking incubator (100 rpm) for 12 h. After incubation, the OD_600_ value of each bacterial culture with phages was measured to indirectly determine their susceptibility to phage infections. Each experiment was performed in triplicate.

## Results

### Isolation, culturing, and morphological characterization of novel *Vibrio* phages

Two novel bacteriophages that infect *V. harveyi* and one that targets *V. parahaemolyticus* were isolated from aquaculture effluents collected from a shrimp farm in Jindo Province, Korea. The isolation process involved environmental sample enrichment followed by serial purification using DLA assays to obtain high-titer, single-genotype lysates. All three phages isolated were capable of actively infecting and lysing their respective host bacteria when co-cultured in MB at 25°C, demonstrating their potential as candidates for phage therapy applications.

**Fig. 1.**
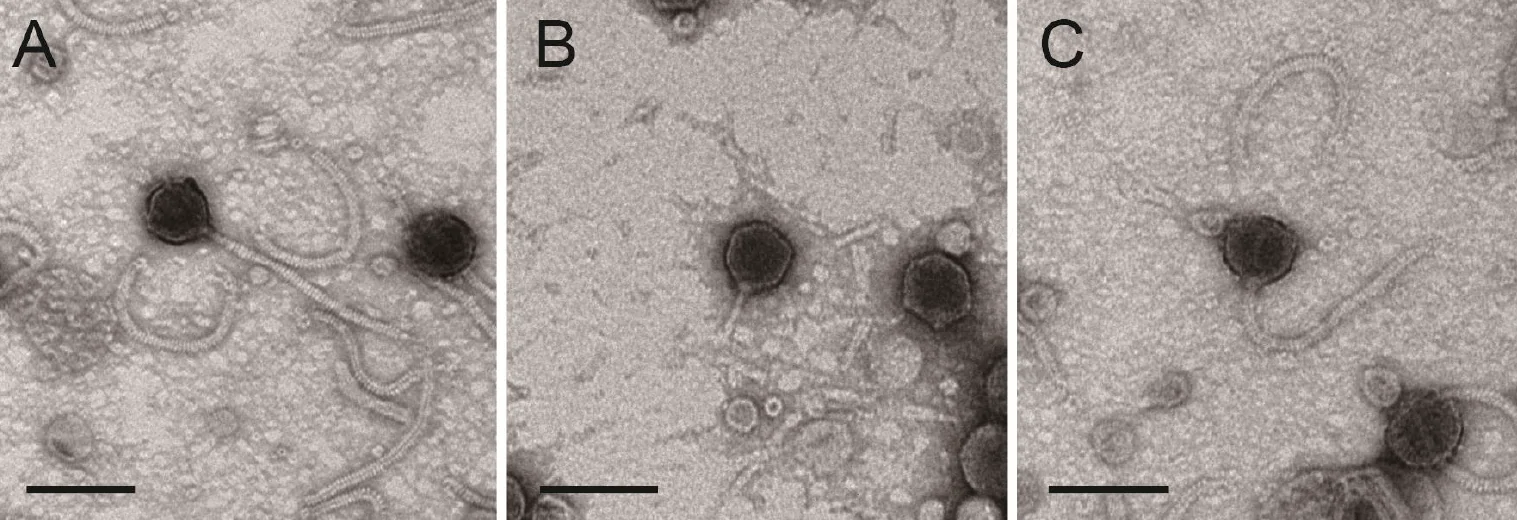
Transmission electron micrographs of the newly isolated *Vibrio* phages negatively stained with 2% uranyl acetate. (A) vB_VhaS-MS01, (B) vB_VpaP-MS02, and (C) vB_VhaS-MS03. Each particle features an icosahedral head and tail structure characteristic of Caudoviricetes. Scale bars: 100 nm.

For morphological taxonomic classification, 200 μl concentrated phage particles were prepared from 200 ml of the bacterial host lysate, and TEM performed thereafter (Fig. 1). TEM analysis revealed that the isolated phages exhibit distinct features similar to those of Caudoviricetes. Specifically, the two phages infecting *V. harveyi* possessed long, contractile tails and icosahedral heads, which are typical morphological characteristics of the *Siphoviridae* family (Fig. 1A and 1C), whereas the phage infecting *V. parahaemolyticus* displayed an icosahedral head with a short tail, consistent with *Podoviridae* morphology (Fig. 1B). Based on these morphological features and host specificity, the phages were designated as vB_VhaS-MS01 and vB_VhaS-MS03 for phages infecting *V. harveyi*, and as vB_VpaP-MS02 for the phage infecting *V. parahaemolyticus* (Adriaenssens and Brister, 2017). Quantitative TEM measurements indicated that vB_VhaS-MS01 had an average head diameter of 59.77 nm (± 3.14 nm) and tail length of 223.61 nm (± 12.75 nm), whereas vB_VhaS-MS03 exhibited a head diameter of 60.21 nm (± 4.82 nm) and tail length of 230.59 nm (22.91 nm). The phage, vB_VpaP-MS02, was found to have an icosahedral head diameter of 57.76 nm (± 1.82 nm) and short tail of 46.70 nm (± 6.36 nm). The quantitative morphological data are summarized in Table 1.

### Genomic analysis and phylogenetic relationships of *Vibrio* phages

Genomic DNA from the three isolated *Vibrio* phages (vB_VhaS-MS01, vB_VpaP-MS02, and vB_VhaS-MS03) was extracted and sequenced on an Illumina HiSeq platform, generating 1 Gb of 150-bp paired-end reads. The raw reads were quality-filtered, trimmed, and assembled de novo using SPAdes (v3.15.5) (Bankevich et al., 2012). Although the initial assemblies did not yield single, complete circular contigs, manual evaluation of the assembly graphs and overlapping sequences using the Bandage program (Wick et al., 2015) allowed us to construct complete and circularized phage genomes. The genome of vB_VhaS-MS01 was 81,710 bp in length, with a GC content of 46.8%; that of vB_VpaP-MS02 was 76,865 bp in length, with a GC content of 38.5%; and that of vB_VhaS-MS03 was 81,874 bp in length, with GC content of 47.6% (Table 1). ORF prediction and functional annotation performed using Prokka (v1.14.6) (Seemann, 2014) and BLASTp against the NCBI nr database revealed that the genomes encode 125, 102, and 124 ORFs, respectively (Tables S1, S2, and S3).

**Fig. 2.**
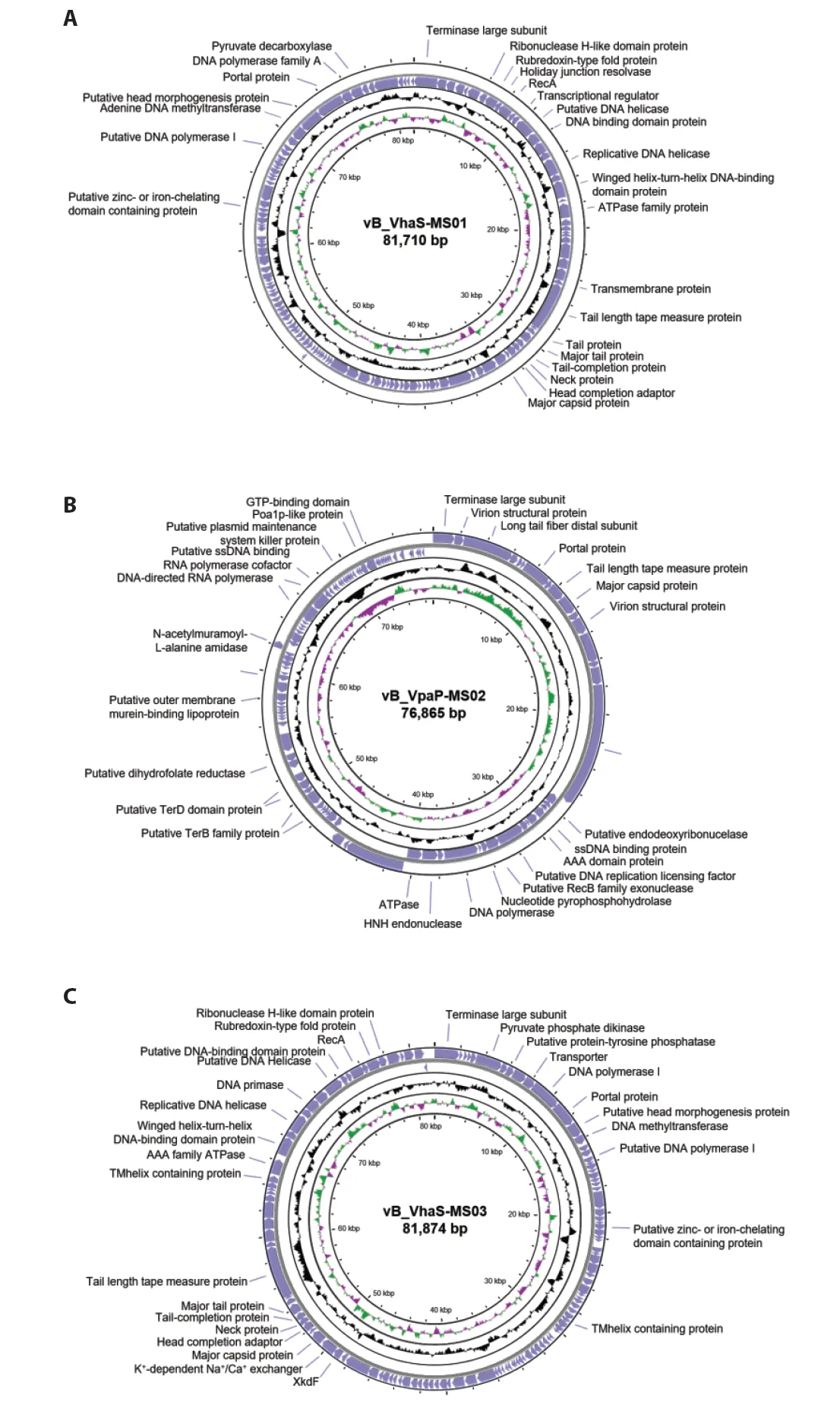
Circular genome maps of the newly isolated *Vibrio* phages generated using the CGView tool. (A) vB_VhaS-MS01, (B) vB_VpaP-MS02, and (C) vB_VhaS-MS03. Each map shows the predicted coding sequences (arrows). The inner ring indicates the GC content and GC skew, and selected genes are labeled to highlight proteins involved in structure formation and other key processes. The annotated circular layouts reveal the modular organization characteristic of tailed phages, underscoring their potential for targeted lytic activity against *Vibrio* pathogens.

Circular genome maps were constructed for each phage to illustrate the overall organization of the predicted ORFs and function annotations (Fig. 2). The outer rings depict the predicted coding sequences, as identified by Prokka and annotated via BLASTp searches. All three genomes displayed a modular organization characteristic of members of the Caudoviricetes, with structural genes encoding head and tail proteins clustered together and adjacent to replication- and lysis-associated regions. Despite this shared overall architecture, several notable differences emerged among the phages. The vB_VhaS-MS01 and vB_VhaS-MS03 phages shared an ANI value of 80.45% (Yoon et al., 2017). ANI measures the average nucleotide-level similarity between two genomes, with higher values suggesting a closer evolutionary relationship. These phages also exhibited a highly similar structural gene organization, reflecting their common host, *V. harveyi*, and similar morphology. Both phages contained distinct modules for terminase, tail fiber, and baseplate proteins, with their *terL* protein sequences sharing 92.69% identity, indicating close evolutionary relatedness (Fig. 2A and 2C). This high sequence identity suggests that these phages share a recent common ancestor and have undergone limited evolutionary divergence. In contrast, the vB_VpaP-MS02 genome displayed a more divergent arrangement, with unique gene clusters that could contribute to its host specificity toward *V. parahaemolyticus* by encoding specialized adsorption or replication mechanisms (Fig. 2B). Furthermore, gene prediction and functional annotation revealed putative proteins associated with DNA polymerases, helicases, and regulatory factors across all three genomes. Notably, while vB_VhaS-MS01 and vB_VhaS-MS03 carry DNA polymerase genes, the vB_VpaP-MS02 phage encodes a DNA-directed RNA polymerase, highlighting its distinctive approach to utilizing host replication machinery. Unlike DNA polymerase, which synthesizes new DNA strands using a DNA template, RNA polymerase transcribes viral genes into RNA, allowing immediate protein synthesis. This independent transcriptional capability may provide a functional advantage by enabling the phage to bypass host transcriptional regulation, ensuring efficient viral gene expression even when the host transcript is suppressed (McAllister and Raskin, 1993; Sousa et al., 2003). Similar strategies have been observed in other *Vibrio* phages, suggesting that this is an evolutionary adaptation for efficient viral replication (Liu et al., 2014). Importantly, none of the genomes encoded known virulence factors, antibiotic-resistance determinants, or integrase genes, suggesting their potential suitability for targeted phage therapy, pending further in vivo validation. Collectively, these novel *Vibrio* phages demonstrated distinct genomic characteristics, with vB_VhaS-mS01 and vB_VhaS-MS03 exhibiting nearly identical genetic architectures, whereas vB_VpaP-MS02 displayed divergence, likely reflecting its unique host specificity.

**Fig. 3.**
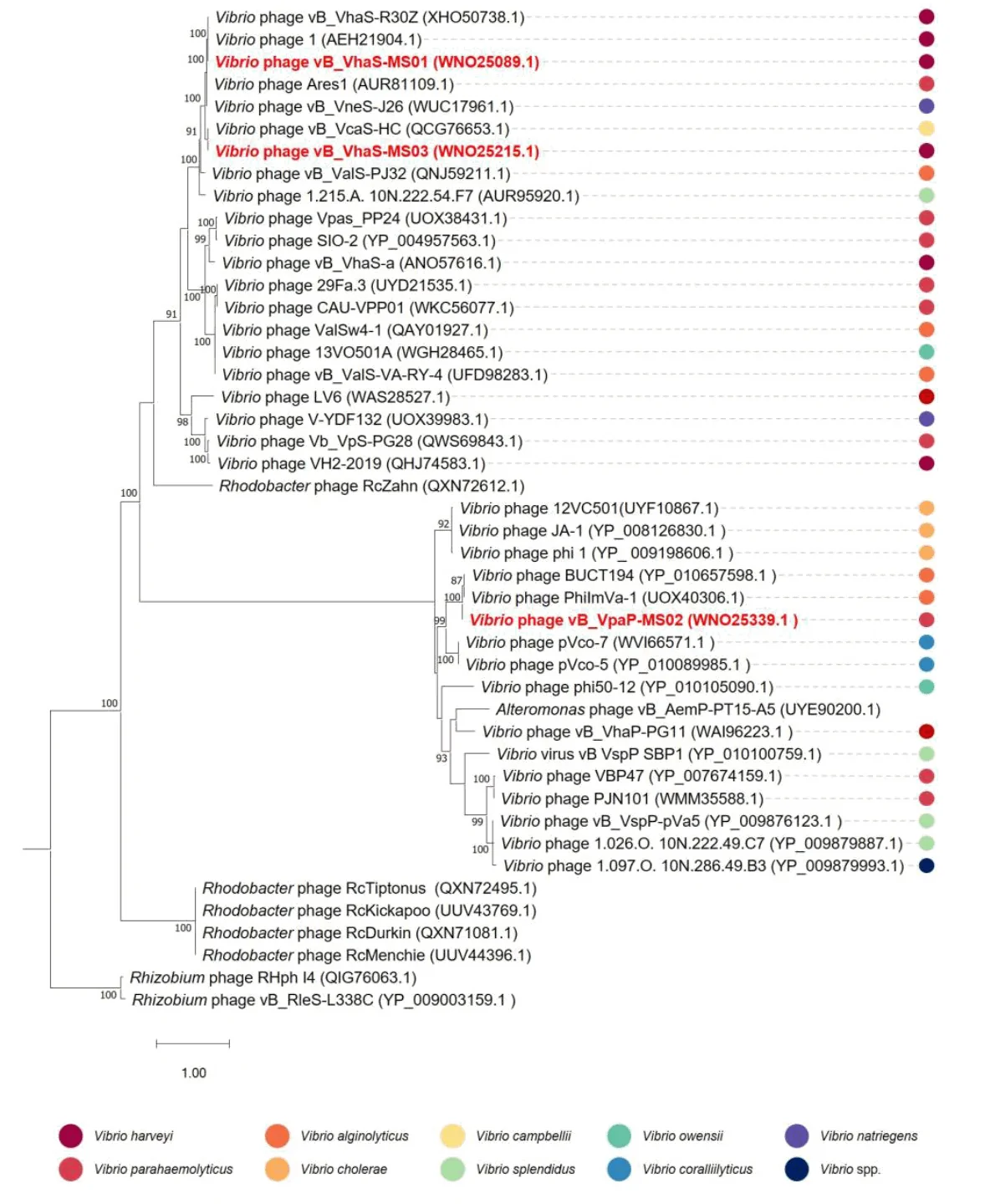
Maximum-likelihood phylogenetic tree of the newly isolated *Vibrio* phages (vB_VhaS-MS01, vB_VhaS-MS03, and vB_VpaP-MS02) and representative phages that infect various *Vibrio* species, as inferred from the terminase large subunit gene (*terL*). Bootstrap values (1,000 replicates) are shown on the nodes, and the color-coded circles indicate host bacteria. The novel phages isolated this study are labeled in red.

To investigate the evolutionary relationships among the newly isolated phages and other known *Vibrio*-infecting phages, a maximum-likelihood phylogenetic tree was constructed based on the *terL* gene (Fig. 3), which is the most well-known conserved phage gene. Bootstrap values for key nodes are indicated in the phylogenetic tree (e.g., the major clade containing vB_VhaS-MS01 and vB_VhaS-MS03 received 97% support, whereas vB_VpaP-MS02 clustered separately with 92% support). These values indicated high confidence in phylogenetic placement of the phages. The tree revealed distinct clades corresponding to phages infecting different *Vibrio* species, as indicated by the color-coded circles denoting host bacteria. The vB_VhaS-MS01 phage clustered closely with other phages that infect *V. harveyi*, forming a highly supported subclade that reflects their shared host range. Although vB_VhaS-MS03 also targets *V. harveyi*, it clustered with other phages that exhibit diverse host ranges, suggesting that vB_VhaS-MS01 and vB_VhaS-MS03 might have undergone unique genetic and evolutionary divergence that distinguishes them from other *Vibrio*-infecting phages. Additionally, vB_VpaP-MS02 formed a distinct branch with phages that infect various *Vibrio* species. Notably, although vB_VpaP-MS02 clustered closely with phages that infect *V. alginolyticus*, it remained clearly distinct from them, indicating that it harbors a novel *terL* gene and thus likely represents a new phage species.

**Fig. 4.**
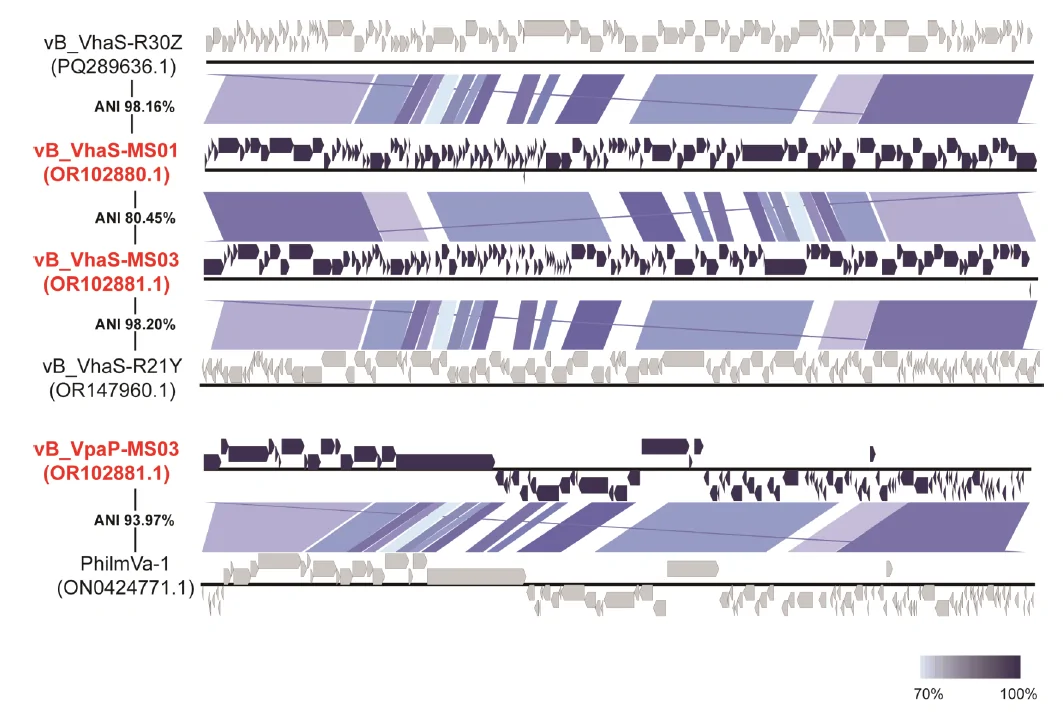
Comparative genomic analysis of the three newly isolated *Vibrio* phages (vB_VhaS-MS01, vB_VpaP-MS02, and vB_VhaS-MS03) alongside their closest known phage relatives, as determined via BLAST similarity and average nucleotide identity (ANI) values. Darker shading between genomes indicates higher sequence homology (70–100% identity). The ANI values are listed for each pairwise comparison.

Furthermore, a comparative genomic analysis was performed between the novel phages and the previously reported and closely related phages (Fig. 4). Regions of high nucleotide similarity (shown as darker-colored blocks) were interspersed with segments that exhibited lower sequence identity or divergent gene arrangements. Specifically, vB_VhaS-MS01 shared an ANI value of 98.16% with vB_VhaS-R30Z, indicating substantial genomic similarity, yet it exhibited only 80.45% ANI with vB_VhaS-MS03, which in turn showed 98.20% ANI with vB_VhaS-R21Y. Both vB_VhaS-R30Z and vB_VhaS-R21Y infect *V. harveyi*, suggesting that while the novel phages share high genomic similarity with those targeting the same host, vB_VhaS-MS01 and vB_VhaS-MS03 follow distinct evolutionary pathways. Additionally, vB_VpaP-MS02 exhibited 93.97% ANI with *Vibrio* phage PhilmVa-1, which infects *V. alginolyticus*. Despite this relatively high sequence conservation, large genomic blocks appeared rearranged or absent in the alignment between these two phages, implying divergent evolutionary trajectories, particularly in the structural and regulatory regions. Taken together, although the novel isolates resembled their known relatives in several respects, their ANI values and syntenic patterns clearly distinguished them. These findings, in conjunction with the phylogenetic and morphological observations, indicate that vB_VhaS-MS01, vB_VhaS-MS03, and vB_VpaP-MS02 follow unique evolutionary routes and represent promising new candidates for controlling pathogenic *Vibrio* species.

### One-step growth curve analysis and burst size determination

**Fig. 5.**
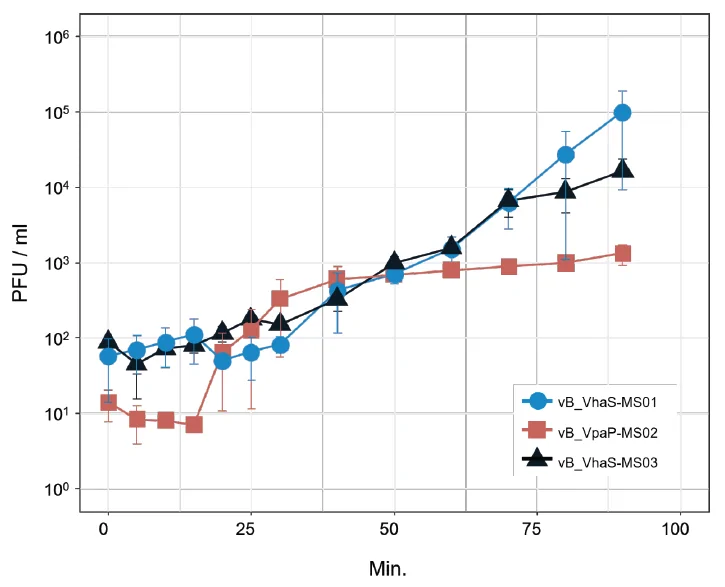
One-step growth of the *Vibrio* phages, vB_VhaS-MS01 (blue circles), vB_VpaP-MS02 (red squares), and vB_VhaS-MS03 (black triangles), showing plaque-forming unit (PFU) counts over a 100-min incubation period at 25°C. Each time point represents the mean of triplicate assays, with error bars indicating the standard deviation. The distinct latent periods, rise phases, and final PFU titers highlight differences in replication dynamics and burst sizes among the three phages.

To determine the replication dynamics and lytic capacities of the newly isolated phages, one-step growth curves were constructed by quantifying plaque-forming units (PFUs) over time (Fig. 5). Each phage was tested in triplicate at 25°C, and the resulting data plotted to determine the latent period, exponential phase, and burst size. Overall, all three phages exhibited active lytic life cycles but demonstrated distinct growth patterns. However, the adsorption rate, which is a critical factor for phage efficacy, was not directly measured in this study. Future studies should assess adsorption kinetics to further elucidate the infection dynamics of these phages. Phages vB_VhaS-MS01 and vB_VhaS-MS03, which both target *V. harveyi*, showed latent periods of approximately 30 min, followed by a gradual increase in PFU titers indicative of a steady exponential phase. At 90 min post-infection, vB_VhaS-MS01 and vB_VhaS-MS03 reached burst sizes of approximately 126 and 5,525 PFU/ml, respectively (n = 3). In contrast, vB_VpaP-MS02, which infects *V. parahaemolyticus*, displayed a shorter latent period of 15 min and more rapid exponential phase, with PFU titers peaking at 60 min. This quicker lytic cycle yielded an average burst size of 94 PFU/ml.

The differences in growth kinetics and final burst sizes among the phages may reflect phage-specific adaptations, such as variations in adsorption efficiency, genome packaging, or lysis mechanisms. Interestingly, the shorter latent period of vB_VpaP-MS02 could facilitate faster bacterial clearance, whereas the higher burst size of vB_VhaS-MS01 might offer greater amplification within a single infection cycle. Taken together, these data underscore the distinct infection strategies of the three novel phages and highlight their potential utility as targeted biocontrol agents in aquaculture settings.

### Multiplicity of infection optimization

**Fig. 6.**
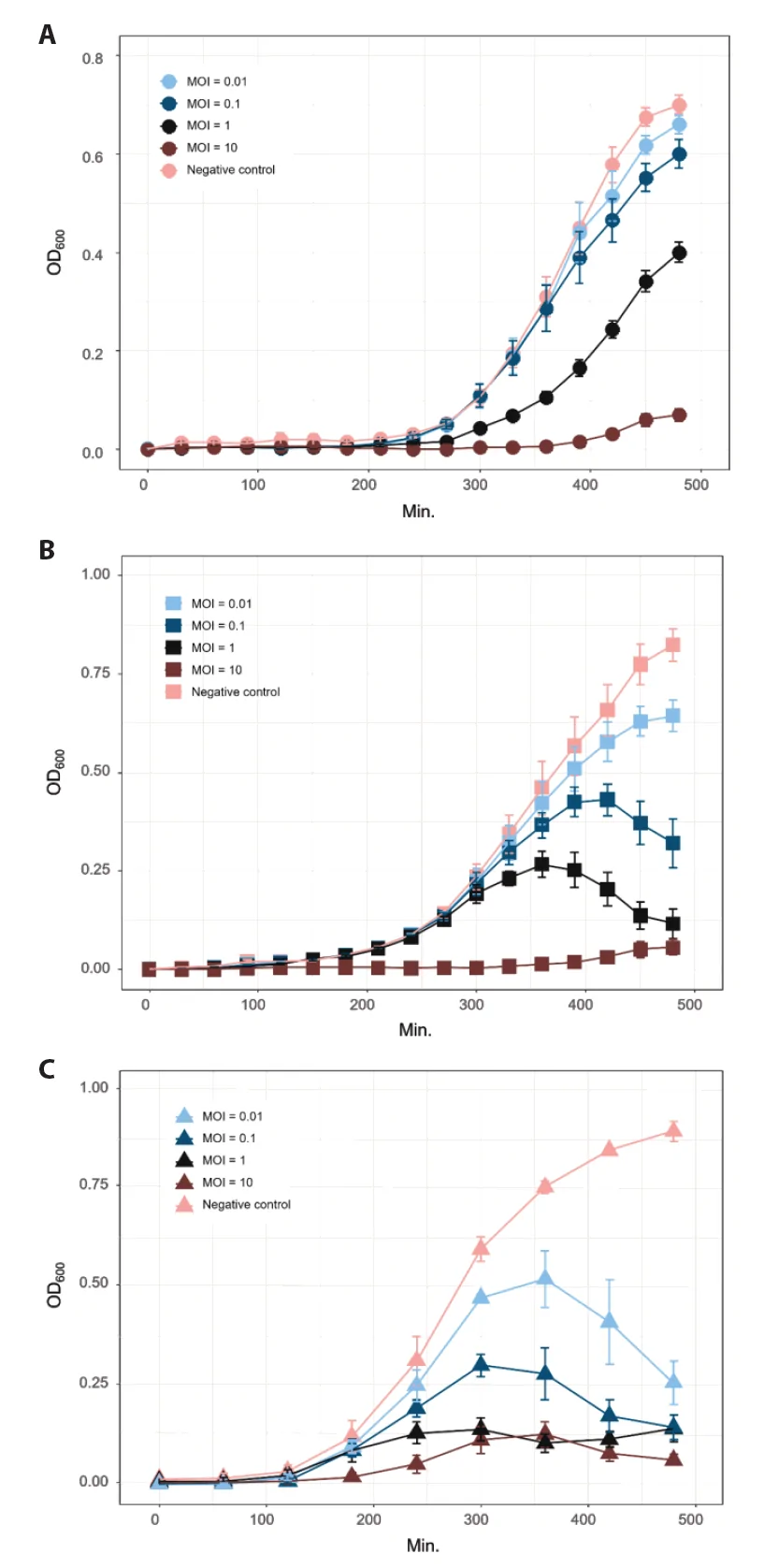
Multiplicity of infection (MOI) optimization for the *Vibrio* phages. Growth curves of each *Vibrio* host (monitored via optical density measurements at 600 nm [OD_600_]) are shown for (A) vB_VhaS-MS01, (B) vB_VpaP-MS02, and (C) vB_VhaS-MS03 at MOIs of 0.01, 0.1, 1, and 10, alongside a negative control (no phages). Each data point represents the mean of triplicate measurements, with error bars indicating standard error.

To determine an effective yet resource-efficient inoculation concentration for phage therapy, we evaluated a range of MOIs for each newly isolated *Vibrio* phage. Phage-host co-cultures were established at MOIs of 0.01, 0.1, 1, and 10, and bacterial growth inhibition monitored via optical density measurements (Fig. 6). Overall, higher MOIs (1 and 10) resulted in a faster and more pronounced suppression of *Vibrio* growth.

For vB_VhaS-MS01, low MOIs (0.01 and 0.1) resulted in moderate suppression of host growth, whereas MOIs of 1 and 10 led to significant inhibition throughout the co-culture period. At an MOI of 10, bacterial growth was completely suppressed, with minimal bacterial proliferation detected at the later stages (Fig. 6A). For vB_VpaP-MS02, bacterial growth was substantially repressed, even at an MOI of 0.01 when compared to the negative control. At MOIs of 0.1 and 1, the bacterial growth curve showed a marked decline beginning at 330 and 420 min after initiating the co-culture, respectively, indicating that an MOI as low as 0.1 is sufficient for immediate control of host cells. Complete suppression was observed at an MOI of 10 (Fig. 6B).

For the vB_VhaS-MS03 phage, all tested MOIs effectively inhibited host growth. At MOIs of 1 and 10, host growth was largely inhibited throughout the assay, although brief periods of growth were observed before subsequent lysis. At MOIs of 0.1 and 0.01, bacterial growth was successfully controlled, and the final cell densities were approximately 6.7- and 3.5-fold lower than those of the negative control, respectively (Fig. 6C). The ability of vB_VhaS-MS03 to remain effective at low MOIs may be attributable to differences in the timing of phage gene expression, which can influence infection efficiency. Some phages can rapidly activate host translation mechanisms and suppress bacterial defense systems, whereas others exhibit delayed gene expression, leading to reduced replication efficiencies (Howard-Varona et al., 2018). Although vB_VhaS-MS01 and vB_VhaS-mS03 infect the same host, the differences in their infection efficiencies observed in this study suggest that their gene expression dynamics and interactions with host replication machinery and defense suppression mechanisms may differ. Further transcriptomic analyses are necessary to confirm the specific molecular basis of these differences. These results suggest that vB_VhaS-MS03 can achieve effective bacterial control even at a low MOI. Based on these findings, we recommend an MOI of 1 for vB_HvaS-MS01, 0.1 for vB_VpaP-MS02, and 0.01 for vB_VhaS-MS03 for the efficient and economical treatment of *Vibrio* infections in aquaculture systems.

### Host range and infection spectrum analysis

**Fig. 7.**
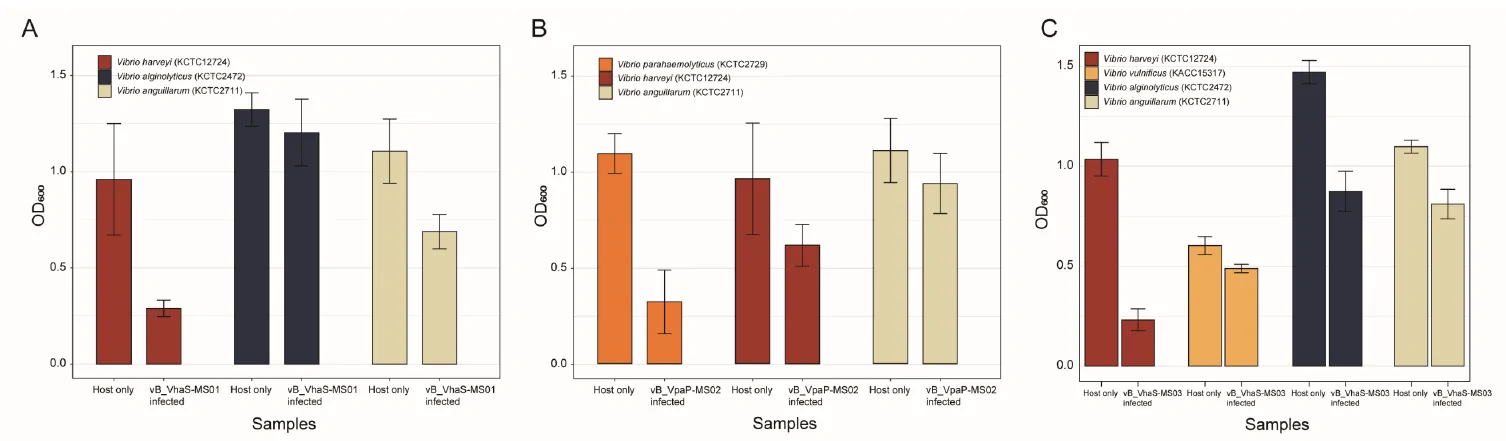
Host range and infection spectrum of the newly isolated *Vibrio* phages. (A) Growth of *V. harveyi* (red bars), *V. alginolyticus* (dark blue bars), and *V. anguillarum* (beige bars) in the presence of vB_VhaS-MS01 (labeled “infected”) versus “Host only” (no phage). (B) Growth of *V. parahamolyticus* (orange bars), *V. harveyi* (red bars), and *V. anguillarum* (beige bars) treated with vB_VpaP-MS02. (C) Growth of *V. harveyi* (red bars), *V. vulnificus* (gold bars), and *V. alginolyticus* (dark blue bars), and *V. anguillarum* (beige bars) exposed to vB_VhaS-MS03. Bars represent the final optical density at 600 nm (OD_600_) values measured after phage treatment (multiplicity of infection [MOI] = 1), showing strong host specificity for each phage. Error bars indicate the standard error of triplicate experiments.

To evaluate whether the newly isolated phages could infect *Vibrio* strains other than their original hosts and thereby potentially broaden the scope of single-phage treatment, we conducted cross-infection assays against several *Vibrio* species commonly implicated in aquaculture diseases (Fig. 7). Each phage was tested against its original host and other clinically relevant pathogens, including *V. alginolyticus*, *V. anguillarum*, and *V. vulnificus*.

The vB_VhaS-MS01 phage, originally isolated from *V. harveyi*, markedly reduced the OD_600_ value of *V. harveyi* cultures and significantly inhibited the growth of *V. anguillarum*. In contrast, it had a minimal effect on *V. alginolyticus*, indicating a relatively narrow host range. The vB_VhaS-MS03 phage exhibited a moderately broader spectrum of activity, effectively suppressing both *V. alginolyticus* and *V. anguillarum*, although its activity against *V. vulnificus* was weak (Fig. 7A). Meanwhile, vB_VpaP-MS02, isolated from *V. parahaemolyticus*, predominantly targeted its original host, with only limited cross-infection observed against other *Vibrio* species (Fig. 7B).

From a therapeutic perspective, the high specificity of these phages minimizes the unintended disruption of nonpathogenic or beneficial microbes in aquaculture systems. However, the narrow host ranges of vB_VhaS-MS01 and vB_VpaP-MS02 suggest that a phage cocktail may be necessary to effectively control mixed infections; vB_VhaS-MS03 demonstrated effective lysis across multiple *Vibrio* species and may serve as a stand-alone therapeutic agent for controlling infections caused by *V. harveyi*, *V. alginolyticus*, and *V. anguillarum* (Fig. 7C). Overall, these results highlight the lytic potency, infection kinetics, and host specificity of the three novel *Vibrio* phages, supporting their potential as safe and targeted alternatives to antibiotics for the management of vibriosis outbreaks in aquaculture.

## Discussion

Effective disease management is critical for the stable operation of aquaculture farms, and current practices rely heavily on antibiotics for both prophylactic and therapeutic purposes (Culot et al., 2019). Although antibiotics offer rapid bacterial suppression, their prolonged and widespread use has led to significant long-term challenges, such as the emergence of multidrug-resistant pathogens (Hossain et al., 2022), accumulation of residual antibiotics in fish and the environment (Su et al., 2024), and consequent disruption of natural microbial communities (Gomes et al., 2020). These issues highlight the urgent need for eco-friendly and sustainable alternatives to conventional antibiotic treatments. Motivated by these challenges, our study focused on developing a region-specific phage therapy approach by isolating and characterizing novel bacteriophages from aquaculture effluents in Korean shrimp farms. Utilizing local resources not only maximizes domestic resource use but also offers a significant advantage over imported phage products. Phages that have coevolved with their target bacteria in the same geographic regions are likely to be better adapted to local environmental conditions and bacterial ecotypes, thereby potentially providing enhanced efficacy for controlling *Vibrio* infections.

In this study, we successfully isolated and characterized three novel *Vibrio* phages from aquaculture effluents in Korean shrimp farms. Our results revealed that vB_VhaS-MS01 and vB_VhaS-MS03, both isolated from *V. harveyi*, exhibited morphological features typical of the *Siphoviridae* family, whereas vB_VpaP-MS02, isolated from *V. parahaemolyticus*, displayed characteristics consistent with those of the *Podoviridae* family. Genomic analyses confirmed that these phages possessed modular genome organizations typical of Caudoviricetes, with vB_VhaS-MS01 and vB_VhaS-MS03 sharing a high ANI and similar structural gene arrangements, whereas vB_VpaP-MS02 exhibited a distinct genomic architecture that likely underpins its host specificity.

One-step growth assays demonstrated that the phages differed in their replication kinetics; vB_VpaP-MS02 had a shorter latent period and rapid exponential phase, whereas vB_VhaS-MS01 and vB_VhaS-MS03 exhibited longer latent periods but with varied burst sizes. Moreover, our MOI experiments revealed that although higher MOIs generally achieved faster and more complete bacterial suppression, vB_VhaS-MS03 could effectively control bacterial growth at a very low MOI of 0.01. These differences in MOIs observed among the phages can be attributed to several factors, including the host bacterial growth rate, phage adsorption efficiency, and replication kinetics. Previous studies have shown that a more rapid adsorption and shorter latent period can enable certain phages to outcompete others when co-cultivated in a liquid medium (Kornienko et al., 2020). In line with this, despite infecting the same host, vB_VhaS-MS01 and vB_VhaS-MS03 likely possess different adsorption rates and latent periods, contributing to their distinct MOIs and lytic characteristics. The higher efficiency of vB_VhaS-MS03 at lower MOIs suggests that its replication dynamics may be optimized for rapid and sustained bacterial suppression, making it a promising candidate for applications in phage therapy. In addition, host range analyses showed that each phage maintained a high specificity for its original host, with vB_VhaS-MS03 displaying a broader spectrum of activity by suppressing three additional *Vibrio* species.

These findings are significant for the following reasons. First, the isolation of novel phages from domestic aquaculture effluents demonstrates the value of leveraging local microbial resources. Phages that evolve in the same environment as their target bacteria are likely to be better adapted to the specific bacterial ecotypes found in Korean aquaculture systems. Consequently, a region-specific phage therapy strategy is expected to be more effective than treatments based on phages isolated in foreign regions are (Koskella and Meaden, 2013). Although this study did not experimentally test the infectivity of the isolated phages against pathogenic *Vibrio* strains from other regions, previous studies have demonstrated that phages with similar host ranges exhibit region-specific ecotypes (Feng et al., 2024). Based on these findings, we hypothesize that the three newly isolated phages from Korea may exhibit higher infection rates against *Vibrio* strains that cause vibriosis in Korea than against strains from other regions. Second, although phage therapy is recognized for its eco-friendly nature and safety—particularly given that these phages do not encode known virulence factors, antibiotic-resistance determinants, or integrase genes—the slower onset of antibacterial action and higher associated costs have historically limited its commercial adoption when compared to antibiotics (Leptihn and Loh, 2022). Unlike antibiotics, which can be chemically synthesized and produced at a low cost, phage therapy requires the mass production and distribution of biologically active agents. However, given the increasing environmental and public health costs associated with antibiotic use, investing in phage therapy may prove economically advantageous in the long term. However, in an era where environmental sustainability and reduction in antibiotic usage are increasingly critical, the development of phage therapy offers a promising alternative. One strategy for overcoming the inherent challenges of phage therapy is to develop phage cocktails that combine multiple phages with complementary host ranges (Costa et al., 2024). Such cocktails would not only broaden the spectrum of bacterial targets but also mitigate the risk of resistance development by simultaneously attacking multiple bacterial receptors or pathways. For instance, our results indicate that while vB_VhaS-MS01 and vB_VpaP-MS02 were highly specific, vB_VhaS-MS03 exhibited a broader host range, effectively lysing *V. harveyi*, *V. alginolyticus*, and *V. anguillarum*, and could potentially serve as a stand-alone agent or key component in a cocktail designed to target mixed infections. Finally, integration of these phages into a targeted therapeutic regimen could be enhanced by combining phage therapy with reduced doses of antibiotics (Liu et al., 2020). Such combination treatments may achieve a synergistic effect that could minimize the emergence of resistance against either agent, ultimately leading to the more effective and sustainable control of *Vibrio* infections in aquaculture.

Given the urgent need to reduce antibiotic usage, driven by concerns over environmental contamination, the emergence of multidrug-resistant pathogens, and the imperative for sustainable aquaculture practices, alternative treatment strategies must be prioritized. One promising approach is the development of phage cocktails that combine multiple phages with diverse host ranges. Such cocktails would not only broaden the spectrum of bacterial targets but also mitigate the risk of resistance development by simultaneously attacking multiple bacterial receptors or pathways. Alternatively, combining phage therapy with lower doses of antibiotics may offer a synergistic effect, reducing overall antibiotic usage while minimizing the risk of resistance to either treatment.

Recent studies (Cai et al., 2024; Hsu et al., 2024) have demonstrated that phages isolated from local environments exhibit superior efficacy in targeting region-specific bacterial strains compared with those obtained from foreign sources. These findings reinforce our observation that leveraging locally adapted phages can significantly enhance treatment efficacy in aquaculture settings (Kalatzis et al., 2023). Phages coevolve with their host bacteria under local environmental conditions and become fine-tuned to infect specific bacterial populations. Conversely, phages sourced from foreign waters often show limited efficacy when applied in different environmental contexts because they are not adapted to the unique bacterial ecotypes and local conditions of the target area (Hanson et al., 2016). In contrast, our region-specific approach, which leverages phages locally isolated from specific aquaculture systems, has proven to be more effective in targeting *Vibrio* strains endemic to the specific environment (Koskella and Brockhurst, 2014). This regional specificity not only enhances therapeutic potential but also minimizes off-target effects, thereby preserving beneficial microbiota. Ultimately, tailoring phage therapy to local bacterial populations is crucial for overcoming the limitations of imported phage products and offering a more effective and sustainable strategy for managing diseases in aquaculture.

Despite the promising in vitro results, several limitations remain. In vivo efficacy of these phages must be validated in aquaculture settings, and the long-term stability of phage formulations under field conditions requires further investigation (Nakai and Park, 2002). Additionally, the potential of phage-resistant bacterial strains developing over prolonged use should be considered (Doss et al., 2017). Beyond these study-specific limitations, phage therapy faces broader challenges, including difficulties in large-scale production, a short shelf-life, and regulatory hurdles for clinical and aquaculture applications (Leptihn and Loh, 2022; Sieiro et al., 2020). Further studies should focus on optimizing phage cocktail formulations and assessing their performance in combination with reduced antibiotic dosages to mitigate resistance and enhance therapeutic outcomes.

In this context, the three phages characterized in our study present a promising opportunity for utilization in phage therapy. Our data indicate that when used in combination, these phages have the potential to form an effective cocktail capable of simultaneously targeting *V. harveyi*, *V. parahaemolyticus*, *V. alginolyticus*, *V. anguillarum*, and *V. vulnificus*. Such a formulation would offer a comprehensive solution for managing mixed *Vibrio* infections, thereby enhancing treatment efficacy and environmental sustainability. Moreover, the broader host range exhibited by vB_VhaS-MS03 suggests that it could serve as either a key component of a cocktail or as a stand-alone agent for controlling multiple *Vibrio* pathogens. In summary, our findings provide valuable insights into the development of targeted, region-specific phage therapy for Korean aquaculture. By harnessing locally sourced phages, we have moved closer to implementing eco-friendly and effective alternatives to antibiotics, a critical step toward sustainable disease management that addresses both industrial needs and environmental preservation.

## Figures and Tables

**Table 1.** General characteristics and genomic information of phages vB_VhaS-MS01, vB_VpaP-MS02, and vB_VhaS-MS03

Bacteriophage	vB_VhaS-MS01	vB_VpaP-MS02	vB_VhaS-MS03
Bacterial host	*Vibrio harveyi*	*Vibrio parahaemolyticus*	*Vibrio harveyi*
Morphology	Siphoviridae	Podoviridae	Siphoviridae
Burst size	126.38 PFU/ml	94.76 PFU/ml	5,525.32 PFU/ml
Genome size	81,710 bp	76,865 bp	81,874 bp
G+C content	46.8%	38.5%	47.6%
No. of CDS	126	102	124
Accession no.	OR102880	OR102882	OR102881

## Supplementary Information

Table S1. Genome annotation of *Vibrio* phage vB_VhaS-MS01

Table S2. Genome annotation of *Vibrio* phage vB_VpaP-MS02

Table S3. Genome annotation of *Vibrio* phage vB_VhaS-MS03
