# Supplementary material for: Characterization of novel bacteriophages for effective phage therapy against Vibrio infections in aquaculture
Source: J Microbiol. 2025 May 27;63(5):e2502009. doi: 10.71150/jm.2502009 (PMC13577077; doi:10.71150/jm.2502009)
Supplement: Supplementary file 1 [file jm-2502009-Supplementary-Table-S1.pdf]

Table S1. Genome annotation of *Vibrio* phage vB_VhaS-MS01

Start	Stop	Strand	Predicted protein name	Closely related species	Query Coverage	E-value	Percent Identity	Accession no.
1983	1	-	Terminase large subunit	Vibrio phage vB_VneS_J26	0.99	0.0E+00	0.98	WYC17961.1
2779	1970	-	Hypothetical protein	Vibrio phage 1	1.00	0.0E+00	1.00	AEH21903.1
3521	2790	-	WbqC-like family protein	Vibrio phage vB_VneS_J26	1.00	2.0E-162	0.94	WYC17959.1
4343	3600	-	Hypothetical protein	Vibrio phage 1	1.00	0.0E+00	1.00	AAS84209.1
4659	4369	-	Hypothetical protein	Vibrio phage 1	1.00	8.0E-63	0.98	AAS84210.1
5701	4724	-	Hypothetical protein	Vibrio phage 1	1.00	0.0E+00	1.00	AEH21899.1
6363	5803	-	Ribonuclease H-like domain protein	Vibrio phage D4	0.98	2.0E-125	0.93	UHD87223.1
7359	6364	-	Rubredoxin-type fold protein	Vibrio phage vB_VneS_J26	1.00	0.0E+00	0.96	WYC17954.1
7762	7349	-	Holliday junction resolvase	Vibrio phage vB_VneS_J26	1.00	2.0E-91	0.96	WYC17953.1
8819	7743	-	RecA protein	Vibrio phage Ares1	1.00	0.0E+00	0.97	AUR81122.1
9222	8833	-	Hypothetical protein	Vibrio phage 1	1.00	3.0E-91	1.00	AEH21894.1
9682	9230	-	Transcriptional regulator	Vibrio phage vB_VneS_J26	1.00	1.0E-91	0.85	WYC17950.1
11205	9778	-	Putative DNA helicase	Vibrio phage vB_VneS_J26	1.00	0.0E+00	0.97	WYC17949.1
12182	11253	-	DNA binding domain protein	Vibrio phage vB_VneS_J26	1.00	0.0E+00	0.96	WYC17948.1
13179	12193	-	Hypothetical protein	Vibrio phage 1	0.98	0.0E+00	1.00	AEH21890.1
13756	13172	-	Hypothetical protein	Vibrio phage Ares1	1.00	5.0E-108	0.84	AUR81143.1
15277	13829	-	Replicative DNA helicase	Vibrio phage Ares1	1.00	0.0E+00	0.96	AUR81114.1
15855	15262	-	Hypothetical protein	Vibrio phage Ares1	1.00	7.0E-137	0.93	AUR81142.1
17345	15855	-	Winged helix-turn-helix DNA-binding domain protein	Vibrio phage vB_VneS_J26	0.99	0.0E+00	0.93	WYC17943.1
17645	17478	-	No significant similarity found					
19059	17839	-	ATPase family protein	Vibrio phage vB_VneS_J26	1.00	0.0E+00	0.94	WYC17942.1
19721	19182	-	TMhelix containing protein	Vibrio phage vB_VneS_J26	1.00	2.0E-117	0.92	WYC17941.1

20158	19724	-	Hypothetical protein	Vibrio phage 1	0.93	2.0E-95	0.99	AEH21882.1
20375	20148	-	Hypothetical protein	Vibrio phage 1	1.00	2.0E-44	1.00	AEH21881.1
20674	20372	-	Hypothetical protein	Vibrio phage 1	1.00	5.0E-66	1.00	AEH21880.1
21199	20675	-	Hypothetical protein	Vibrio phage 1	1.00	9.0E-121	1.00	AEH21879.1
22500	21202	-	Hypothetical protein	Vibrio phage 1	1.00	0.0E+00	1.00	AEH21878.1
23396	22512	-	Hypothetical protein	Vibrio phage 1	1.00	0.0E+00	0.99	AEH21877.1
24397	23408	-	Hypothetical protein	Vibrio phage 1	1.00	0.0E+00	1.00	AEH21876.1
24794	24408	-	Transmembrane protein	Vibrio phage vB_VneS_J26	1.00	1.0E-79	0.91	WYC17933.1
28930	24791	-	Tail length tape measure protein	Vibrio phage vB_VneS_J26	1.00	0.0E+00	0.92	WYC17932.1
29060	28938	-	Hypothetical protein	Vibrio phage 1	1.00	7.0E-19	1.00	AEH21873.1
29560	29138	-	Tail protein	Vibrio phage vB_VneS_J26	1.00	4.0E-88	0.94	WYC17931.1
30444	29650	-	Major tail protein	Vibrio phage vB_VneS_J26	1.00	0.0E+00	0.95	WYC17930.1
30989	30498	-	Tail-completion protein	Vibrio phage vB_VneS_J26	1.00	5.0E-108	0.90	WYC17929.1
31450	30986	-	Neck protein	Vibrio phage vB_VneS_J26	1.00	5.0E-104	0.97	WYC17928.1
32085	31447	-	Head completion adaptor	Vibrio phage vB_VneS_J26	1.00	7.0E-125	0.87	WYC17927.1
32384	32097	-	Hypothetical protein	Vibrio phage 1	1.00	4.0E-62	1.00	AEH21867.1
33410	32460	-	Major capsid protein	Vibrio phage Ares1	1.00	0.0E+00	0.97	AUR81128.1
34678	33497	-	Hypothetical protein	Vibrio phage 1	1.00	0.0E+00	0.96	AEH21865.1
35292	34681	-	XkdF	Vibrio phage vB_VcaS_HC	1.00	7.0E-131	0.89	QCG76691.1
35771	35421	-	Hypothetical protein	Vibrio phage 1	1.00	2.0E-73	0.97	AEH21863.1
37807	35837	-	Hypothetical protein	Vibrio phage vB_VhaS_R21Y	1.00	0.0E+00	0.98	WKV32815.1
38588	37800	-	Coil containing protein	Vibrio phage vB_VcaS_HC	1.00	0.0E+00	0.95	QCG76693.1
38931	38659	-	Hypothetical protein	Vibrio phage 1	1.00	1.0E-59	1.00	AEH21860.1
39455	38931	-	Hypothetical protein	Vibrio phage 1	1.00	6.0E-123	0.97	AEH21859.1
39769	39467	-	Hypothetical protein	Vibrio phage vB_VneS_J26	1.00	6.0E-63	0.94	WYC17917.1

40069	39779	-	Hypothetical protein	Vibrio phage 1	1.00	1.0E-61	0.99	AEH21857.1
40469	40203	-	Hypothetical protein	Vibrio phage 1	1.00	5.0E-53	0.95	AEH21856.1
41165	40479	-	Hypothetical protein	Vibrio phage 1	1.00	4.0E-160	0.98	AEH21855.1
41666	41190	-	Hypothetical protein	Vibrio phage 1	1.00	1.0E-111	0.99	AEH21854.1
42017	41679	-	Hypothetical protein	Vibrio phage 1	1.00	2.0E-75	1.00	AEH21853.1
42513	42088	-	Hypothetical protein	Vibrio phage 1	1.00	7.0E-101	1.00	AEH21852.1
42896	42510	-	Hypothetical protein	Vibrio phage 1	1.00	8.0E-84	0.98	AEH21851.1
43146	42898	-	Hypothetical protein	Vibrio phage Ares1	1.00	1.0E-41	0.83	AUR81209.1
43558	43196	-	Hypothetical protein	Vibrio phage 1	1.00	2.0E-80	0.99	AEH21849.1
44217	43633	-	Coil containing protein	Vibrio phage D4	1.00	1.0E-128	0.91	UHD87302.1
44509	44195	-	Hypothetical protein	Vibrio phage D4	1.00	2.0E-70	1.00	UHD87301.1
45605	44640	-	Hypothetical protein	Vibrio phage vB_VcaS_HC	1.00	0.0E+00	0.90	QCG76707.1
46671	45679	-	Hypothetical protein	Vibrio phage D4	0.90	1.0E-178	0.93	UHD87299.1
48210	46684	-	Hypothetical protein	Vibrio phage 1	1.00	0.0E+00	0.98	AEH21844.1
48476	48207	-	TMhelix containing protein	Vibrio phage vB_VneS_J26	1.00	2.0E-38	0.78	WYC17900.1
48745	48473	-	Hypothetical protein	Vibrio phage Ares1	0.98	3.0E-39	0.74	AUR81205.1
48996	48796	-	Hypothetical protein	Vibrio phage 1	1.00	3.0E-37	1.00	AEH21843.1
49262	49119	-	Hypothetical protein	Vibrio phage 1	1.00	2.0E-25	1.00	AEH21842.1
49462	49265	-	Hypothetical protein	Vibrio phage 1	1.00	2.0E-39	1.00	AEH21841.1
49711	49475	-	Hypothetical protein	Vibrio phage 1	1.00	9.0E-51	1.00	AEH21840.1
50020	49715	-	Coil containing protein	Vibrio phage vB_VcaS_HC	1.00	4.0E-66	0.98	QCG76714.1
50262	50062	-	Hypothetical protein	Vibrio phage 1	1.00	1.0E-38	1.00	AEH21838.1
50305	50418	+	No significant similarity found					
50757	50395	-	Hypothetical protein	Vibrio phage 1	1.00	4.0E-79	0.99	AEH21837.1
51181	50747	-	Hypothetical protein	Vibrio phage 1	1.00	7.0E-102	1.00	AEH21836.1

51570	51235	-	Hypothetical protein	Vibrio phage 1	1.00	5.0E-72	1.00	AEH21835.1
51960	51628	-	Hypothetical protein	Vibrio phage 1	1.00	2.0E-71	0.98	AEH21834.1
52255	52076	-	Hypothetical protein	Vibrio phage D4	0.93	9.0E-21	0.76	UHD87279.1
52613	52248	-	Hypothetical protein	Vibrio phage 1	1.00	3.0E-82	0.98	AEH21833.1
52996	52610	-	Hypothetical protein	Vibrio phage 1	1.00	4.0E-86	0.98	AEH21832.1
53297	53046	-	Hypothetical protein	Vibrio phage 1	1.00	2.0E-53	1.00	AEH21831.1
53853	53437	-	Hypothetical protein	Vibrio phage 1	1.00	1.0E-96	1.00	AEH21830.1
54340	53840	-	Hypothetical protein	Vibrio phage 1	1.00	1.0E-117	0.99	AEH21829.1
54714	54346	-	Hypothetical protein	Vibrio phage 1	1.00	2.0E-84	1.00	AEH21828.1
55201	54749	-	Hypothetical protein	Vibrio phage D4	0.99	2.0E-92	0.83	UHD87275.1
55632	55252	-	Hypothetical protein	Vibrio phage 1	1.00	2.0E-85	0.99	AEH21826.1
56111	55713	-	Hypothetical protein	Vibrio phage 1	1.00	2.0E-93	0.97	AEH21825.1
56359	56117	-	Hypothetical protein	Vibrio phage 1	1.00	3.0E-49	0.99	AEH21824.1
56776	56432	-	Hypothetical protein	Vibrio phage 1	1.00	3.0E-75	0.96	AEH21823.1
56985	56791	-	Hypothetical protein	Vibrio phage 1	1.00	5.0E-35	0.95	AEH21822.1
57264	57106	-	Hypothetical protein	Vibrio phage D4	1.00	7.0E-10	0.56	UHD87270.1
57847	57248	-	Hypothetical protein	Vibrio phage 1	1.00	4.0E-142	0.96	AEH21821.1
58414	57908	-	Hypothetical protein	Vibrio phage 1	1.00	1.0E-111	0.96	AAL87204.1
59343	58546	-	Hypothetical protein	Vibrio phage 1	1.00	0.0E+00	0.97	AEH21818.1
60055	59330	-	Hypothetical protein	Vibrio phage 1	1.00	9.0E-173	0.98	AEH21817.1
60456	60052	-	Hypothetical protein	Vibrio phage 1	1.00	4.0E-96	1.00	AAL85362.1
60634	60458	-	Hypothetical protein	Vibrio phage 1	1.00	1.0E-32	0.98	AEH21815.1
61053	60622	-	Hypothetical protein	Vibrio phage D4	0.99	2.0E-77	0.80	UHD87262.1
61664	61530	-	Hypothetical protein	Vibrio phage 1	1.00	7.0E-20	0.95	AEH21938.1
61848	61669	-	Hypothetical protein	Vibrio phage 1	1.00	7.0E-33	1.00	AEH21937.1

62096	61848	-	Hypothetical protein	Vibrio phage 1	1.00	2.0E-49	0.95	AEH21936.1
62620	62096	-	Hypothetical protein	Vibrio phage 1	0.95	3.0E-119	0.99	AEH21935.1
63065	62607	-	Putative zinc- or iron-chelating domain containing protein	Vibrio phage vB_VcaS_HC	0.98	4.0E-96	0.90	QCG76740.1
63440	63075	-	Hypothetical protein	Vibrio phage 1	1.00	4.0E-80	0.98	AEH21933.1
64065	63466	-	Hypothetical protein	Vibrio phage vB_VhaS_R21Y	1.00	3.0E-86	0.65	WKV32881.1
65448	64111	-	Hypothetical protein	Vibrio phage D4	0.97	0.0E+00	0.83	UHD87254.1
65808	65500	-	Hypothetical protein	Vibrio phage 1	1.00	2.0E-67	0.98	AEH21930.1
66192	65893	-	Hypothetical protein	Vibrio phage D4	1.00	1.0E-63	0.98	UHD87252.1
66482	66198	-	Hypothetical protein	Vibrio phage vB_VneS_J26	1.00	3.0E-57	0.91	WYC17981.1
66973	66485	-	Hypothetical protein	Vibrio phage vB_VhaS_R21Y	1.00	6.0E-75	0.68	WKV32875.1
67234	66977	-	Hypothetical protein	Vibrio phage vB_ValS_PJ32	0.98	6.0E-21	0.69	QNJ59117.1
67657	67238	-	Hypothetical protein	Vibrio phage 1	0.92	1.0E-85	0.98	AEH21925.1
67823	67638	-	Hypothetical protein	Vibrio phage 1	1.00	1.0E-36	1.00	AEH21924.1
68194	67826	-	Putative DNA polymerase I	Vibrio phage vB_VneS_J26	0.99	9.0E-67	0.75	WYC17977.1
68774	68319	-	Hypothetical protein	Vibrio phage 1	1.00	1.0E-107	1.00	AEH21922.1
69190	68783	-	TMhelix containing protein	Vibrio phage vB_VneS_J26	1.00	3.0E-86	0.92	WYC17975.1
70023	69373	-	Adenine DNA methyltransferase	Vibrio phage vB_VneS_J26	1.00	3.0E-153	0.95	WYC17974.1
71111	70029	-	Putative head morphogenesis protein	Vibrio phage vB_VneS_J26	1.00	0.0E+00	0.91	WYC17973.1
72985	71108	-	Portal protein	Vibrio phage vB_VneS_J26	1.00	0.0E+00	0.92	WYC17972.1
75344	72987	-	DNA polymerase family A	Vibrio phage vB_VneS_J26	1.00	0.0E+00	0.98	WYC17971.1
76192	75344	-	Pyruvate decarboxylase	Vibrio phage vB_VneS_J26	0.99	3.0E-176	0.84	WYC17970.1
77213	76203	-	Hypothetical protein	Vibrio phage 1	1.00	0.0E+00	1.00	AEH21913.1
77732	77235	-	Putative protein-tyrosine phosphatase	Vibrio phage Ares1	1.00	2.0E-103	0.87	AUR81147.1
78265	77783	-	Hypothetical protein	Vibrio phage 1	1.00	4.0E-107	0.98	AEH21911.1
80345	78270	-	Hypothetical protein	Vibrio phage 1	1.00	0.0E+00	1.00	AEH21910.1

80791	80378	-	Coil containing protein	Vibrio phage vB_VneS_J26	1.00	6.0E-67	0.69	WYC17965.1
81047	80781	-	Hypothetical protein	Vibrio phage 1	1.00	9.0E-56	1.00	AEH21907.1
81355	81092	-	Hypothetical protein	Vibrio phage 1	1.00	4.0E-52	0.99	AEH21906.1
81699	81457	-	Hypothetical protein	Vibrio phage 1	1.00	5.0E-51	1.00	AEH21905.1
