# Supplementary material for: Characterization of novel bacteriophages for effective phage therapy against Vibrio infections in aquaculture
Source: J Microbiol. 2025 May 27;63(5):e2502009. doi: 10.71150/jm.2502009 (PMC13577077; doi:10.71150/jm.2502009)
Supplement: Supplementary file 2 [file jm-2502009-Supplementary-Table-S2.pdf]

Table S2. Genome annotation of *Vibrio* phage vB_VpaP-MS02

Start	Stop	Strand	Predicted protein name	Closely related species	Query Coverage	E-value	Percent Identity	Accession No.
1	1593	+	Terminase large subunit	Vibrio phage PhiImVa-1	1.00	0.0E+00	0.98	UOX40306.1
1596	2282	+	Virion structural protein	Vibrio phage BUCT194	1.00	1.0E-164	0.96	YP_010657597.1
2279	6034	+	Long tail fiber distal subunit	Vibrio phage PhiImVa-1	1.00	0.0E+00	0.84	UOX40304.1
6021	6542	+	Hypothetical protein	Vibrio phage PhiImVa-1	1.00	9.0E-113	0.94	UOX40303.1
6611	6928	+	Hypothetical protein	Vibrio phage PhiImVa-1	1.00	1.0E-65	1.00	UOX40302.1
6925	7161	+	Hypothetical protein	Vibrio phage PhiImVa-1	1.00	1.0E-35	0.92	UOX40301.1
7200	9314	+	Portal protein	Vibrio phage PhiImVa-1	1.00	0.0E+00	0.99	UOX40300.1
9307	9663	+	Hypothetical protein	Vibrio phage PhiImVa-1	1.00	6.0E-75	0.98	UOX40299.1
9667	10821	+	Tail length tape measure protein	Vibrio phage BUCT194	0.99	0.0E+00	0.92	YP_010657591.1
10839	12155	+	Major capsid protein	Vibrio phage PhiImVa-1	1.00	0.0E+00	0.98	UOX40297.1
12207	12704	+	Hypothetical protein	Vibrio phage PhiImVa-1	1.00	4.0E-115	0.98	UOX40296.1
12709	13884	+	Virion structural protein	Vibrio phage BUCT194	1.00	0.0E+00	0.78	YP_010657588.1
13946	16120	+	Hypothetical protein	Vibrio phage PhiImVa-1	1.00	0.0E+00	0.98	UOX40294.1
16117	16515	+	Hypothetical protein	Vibrio phage PhiImVa-1	1.00	6.0E-83	0.95	UOX40293.1
16526	17824	+	Hypothetical protein	Vibrio phage PhiImVa-1	1.00	0.0E+00	0.95	UOX40292.1
17833	27021	+	Virion-encapsulated RNA polymerase	Vibrio phage PhiImVa-1	1.00	0.0E+00	0.96	UOX40291.1
27804	27052	-	Hypothetical protein	Vibrio phage PhiImVa-1	1.00	7.0E-161	0.96	UOX40290.1
28190	27804	-	Putative endodeoxyribonuclease	Vibrio phage PhiImVa-1	0.99	1.0E-85	0.98	UOX40321.1
28346	28221	-	Hypothetical protein	Vibrio phage BUCT194	1.00	1.0E-19	1.00	YP_010657580.1
28467	28336	-	Hypothetical protein	Vibrio phage PhiImVa-1	1.00	3.0E-18	0.91	UOX40288.1
29250	28534	-	ssDNA binding protein	Vibrio phage PhiImVa-1	1.00	6.0E-170	0.97	UOX40287.1
30064	29330	-	AAA domain protein	Vibrio phage BUCT194	1.00	2.0E-179	0.99	YP_010657577.1
30279	30064	-	Hypothetical protein	Vibrio phage PhiImVa-1	1.00	1.0E-37	0.86	UOX40285.1
30812	30276	-	Hypothetical protein	Flavobacteriaceae bacterium	0.96	1.0E-43	0.43	MBP94165.1
32971	30812	-	Putative DNA replication licensing factor	Vibrio phage PhiImVa-1	0.99	0.0E+00	0.98	UOX40284.1
33983	32982	-	Putative RecB family exonuclease	Vibrio phage PhiImVa-1	1.00	0.0E+00	0.99	UOX40283.1
34428	33970	-	Nucleotide pyrophosphohydrolase	Vibrio phage BUCT194	1.00	1.0E-99	0.93	YP_010657573.1

34674	34498	-	Hypothetical protein	Vibrio phage BUCT194	1.00	4.0E-24	0.86	YP_010657572.1
37517	34773	-	DNA polymerase	Vibrio phage PhiImVa-1	1.00	0.0E+00	0.98	UOX40280.1
38023	37583	-	Hypothetical protein	Vibrio phage BUCT194	1.00	9.0E-94	0.93	YP_010657570.1
39292	38069	-	HNH endonuclease	Vibrio phage BUCT194	1.00	0.0E+00	0.99	YP_010657568.1
40506	39343	-	ATPase	Vibrio phage PhiImVa-1	1.00	0.0E+00	0.99	UOX40277.1
40665	45068	+	Hypothetical protein	Vibrio phage 393E50-1	0.83	0.0E+00	0.45	CAH9015432.1
45073	45363	+	Hypothetical protein	Vibrio phage 1.119.O._10N.261.51.A9	0.93	3.0E-35	0.61	AUR88976.1
45540	46379	+	Hypothetical protein	Aeromonas phage vB_AsaP_MQM1	0.37	2.0E-16	0.52	WFZ78492.1
46863	46414	-	Hypothetical protein	Vibrio phage phi 1	1.00	8.0E-51	0.52	YP_009198568.1
47668	46976	-	Hypothetical protein	Vibrio phage PhiImVa-1	1.00	1.0E-145	0.92	UOX40275.1
47842	47678	-	Hypothetical protein	Vibrio phage PhiImVa-1	1.00	3.0E-29	0.98	UOX40274.1
48162	47842	-	Hypothetical protein	Vibrio phage PhiImVa-1	0.99	1.0E-63	0.92	UOX40273.1
48356	48162	-	Hypothetical protein	Vibrio phage PhiImVa-1	1.00	6.0E-36	0.91	UOX40272.1
48831	48406	-	Putative TerB family protein	Vibrio phage PhiImVa-1	1.00	2.0E-91	0.97	UOX40270.1
49954	48935	-	Membrane protein	Vibrio phage BUCT194	1.00	0.0E+00	0.98	YP_010657560.1
50406	49987	-	Putative TerB family protein	Vibrio phage PhiImVa-1	1.00	2.0E-96	0.99	UOX40268.1
51086	50406	-	Putative TerD domain protein	Vibrio phage PhiImVa-1	1.00	6.0E-164	0.99	UOX40267.1
51796	51134	-	TerD domain protein	Vibrio phage BUCT194	1.00	2.0E-160	0.98	YP_010657558.1
52925	52329	-	Hypothetical protein	Vibrio phage BUCT194	1.00	5.0E-126	0.97	YP_010657557.1
53500	52922	-	Putative dihydrofolate reductase	Vibrio phage PhiImVa-1	1.00	3.0E-138	0.97	UOX40320.1
55206	53767	-	Hypothetical protein	Vibrio phage PhiImVa-1	1.00	0.0E+00	0.97	UOX40264.1
56157	55807	-	Hypothetical protein	Photobacterium aquimaris	1.00	2.0E-54	0.68	PSU31180.1
56496	56278	-	Hypothetical protein	Neobacillus sp.	0.72	3.0E-04	0.47	MDF2788630.1
56706	56515	-	Hypothetical protein	Vibrio phage PhiImVa-1	1.00	4.0E-34	0.95	UOX40262.1
57002	56709	-	Hypothetical protein	Vibrio phage PhiImVa-1	1.00	1.0E-60	0.95	UOX40261.1
57154	56999	-	Hypothetical protein	Vibrio phage BUCT194	1.00	5.0E-28	0.96	YP_010657551.1
57431	57147	-	Hypothetical protein	Vibrio phage BUCT194	1.00	5.0E-48	0.86	YP_010657550.1
57772	57479	-	Hypothetical protein	Vibrio phage BUCT194	1.00	9.0E-65	1.00	YP_010657548.1
58458	57781	-	Putative outer membrane murein-binding lipoprotein	Vibrio phage PhiImVa-1	0.94	8.0E-108	0.92	UOX40318.1
59342	58551	-	Hypothetical protein	Vibrio phage PhiImVa-1	1.00	0.0E+00	0.96	UOX40257.1

59928	59461	-	Hypothetical protein	Vibrio phage PhiImVa-1	0.42	2.0E-32	0.86	UOX40256.1
60153	60001	-	Hypothetical protein	Vibrio phage PhiImVa-1	0.98	4.0E-25	0.94	UOX40255.1
60644	60432	-	Hypothetical protein	Vibrio phage PhiImVa-1	1.00	6.0E-40	1.00	UOX40254.1
61075	60686	-	Hypothetical protein	Vibrio phage BUCT194	0.84	4.0E-45	0.61	YP_010657648.1
61520	61035	-	Hypothetical protein	Vibrio phage PhiImVa-1	1.00	1.0E-114	0.98	UOX40253.1
61831	61520	-	Hypothetical protein	Vibrio phage PhiImVa-1	0.98	1.0E-65	0.99	UOX40252.1
61853	62383	+	N-acetylmuramoyl-L-alanine amidase	Vibrio phage PhiImVa-1	1.00	4.0E-123	0.97	UOX40317.1
62736	62380	-	Hypothetical protein	Vibrio phage PhiImVa-1	1.00	2.0E-77	0.93	UOX40251.1
63478	62714	-	Hypothetical protein	Vibrio phage PhiImVa-1	0.27	1.0E-36	0.86	UOX40250.1
63773	63492	-	Hypothetical protein	Vibrio phage PhiImVa-1	1.00	3.0E-60	0.96	UOX40246.1
64052	63849	-	Hypothetical protein	Vibrio phage PhiImVa-1	1.00	7.0E-41	1.00	UOX40245.1
64268	64128	-	Hypothetical protein	Vibrio phage PhiImVa-1	1.00	7.0E-23	0.91	UOX40244.1
64617	64423	-	Hypothetical protein	Vibrio phage PhiImVa-1	1.00	7.0E-35	0.89	UOX40243.1
65916	64669	-	DNA-directed RNA polymerase	Vibrio phage PhiImVa-1	1.00	0.0E+00	0.99	UOX40316.1
67250	65961	-	DNA-directed RNA polymerase	Vibrio phage PhiImVa-1	1.00	0.0E+00	0.91	UOX40315.1
67643	67302	-	Hypothetical protein	Vibrio phage PhiImVa-1	1.00	1.0E-74	0.98	UOX40242.1
67986	67711	-	Putative ssDNA binding RNA polymerase cofactor	Vibrio phage PhiImVa-1	1.00	5.0E-54	0.95	UOX40314.1
68343	68113	-	Hypothetical protein	Vibrio phage PhiImVa-1	1.00	3.0E-47	0.97	UOX40241.1
68516	68343	-	Hypothetical protein	Vibrio phage PhiImVa-1	1.00	2.0E-31	0.98	UOX40240.1
68893	68675	-	Hypothetical protein	Vibrio phage BUCT194	1.00	9.0E-45	0.97	YP_010657624.1
69087	68884	-	Hypothetical protein	Vibrio phage PhiImVa-1	1.00	1.0E-33	0.81	UOX40238.1
69279	69166	-	Hypothetical protein	Vibrio phage PhiImVa-1	1.00	3.0E-14	0.95	UOX40237.1
69501	69289	-	Putative plasmid maintenance system killer protein	Vibrio phage PhiImVa-1	1.00	8.0E-41	0.99	UOX40313.1
69625	69503	-	Hypothetical protein	Vibrio phage PhiImVa-1	1.00	1.0E-18	1.00	UOX40236.1
69821	69615	-	Hypothetical protein	Vibrio phage PhiImVa-1	1.00	2.0E-40	0.99	UOX40235.1
69960	69808	-	Hypothetical protein	Vibrio phage PhiImVa-1	1.00	7.0E-24	1.00	UOX40234.1
70219	70022	-	Hypothetical protein	Vibrio phage PhiImVa-1	1.00	2.0E-39	0.95	UOX40233.1
70583	70386	-	No siginificant similarity found					
70768	70580	-	Hypothetical protein	Vibrio phage BUCT194	1.00	1.0E-35	0.94	YP_010657616.1
71294	70782	-	TPA: Poa1p-like protein	Caudoviricetes sp.	1.00	6.0E-57	0.57	DAK29431.1

72087	71308	-	GTP-binding domain	Vibrio phage BUCT194	1.00	0.0E+00	0.98	YP_010657614.1
72323	72114	-	Hypothetical protein	Vibrio phage PhiImVa-1	0.98	1.0E-33	0.79	UOX40229.1
72474	72310	-	Hypothetical protein	Vibrio phage PhiImVa-1	1.00	8.0E-23	0.82	UOX40228.1
72700	72512	-	No siginificant similarity found					
72890	72672	-	Hypothetical protein	Vibrio phage PhiImVa-1	0.84	4.0E-25	0.75	UOX40226.1
73045	72878	-	Hypothetical protein	Vibrio phage PhiImVa-1	0.96	4.0E-27	0.92	UOX40225.1
73229	73032	-	Hypothetical protein	Vibrio phage BUCT194	0.96	3.0E-31	0.86	YP_010657610.1
73399	73226	-	Hypothetical protein	Vibrio phage PhiImVa-1	1.00	2.0E-23	0.79	UOX40224.1
73898	73587	-	Hypothetical protein	Bacteroidota bacterium	0.61	1.0E-14	0.48	NBP66388.1
74506	74165	-	Hypothetical protein	Vibrio phage PhiImVa-1	1.00	1.0E-74	0.99	UOX40223.1
75090	74971	-	Hypothetical protein	Vibrio phage PhiImVa-1	0.94	6.0E-03	0.50	UOX40222.1
75250	75077	-	Hypothetical protein	Vibrio phage BUCT194	1.00	2.0E-29	0.88	YP_010657604.1
75687	75475	-	Hypothetical protein	Vibrio phage PhiImVa-1	0.94	2.0E-28	0.77	UOX40311.1
75854	75672	-	Hypothetical protein	Vibrio phage PhiImVa-1	1.00	6.0E-35	0.95	UOX40310.1
76137	75949	-	Hypothetical protein	Vibrio phage PhiImVa-1	1.00	1.0E-33	0.97	UOX40309.1
