# Supplementary material for: Characterization of novel bacteriophages for effective phage therapy against Vibrio infections in aquaculture
Source: J Microbiol. 2025 May 27;63(5):e2502009. doi: 10.71150/jm.2502009 (PMC13577077; doi:10.71150/jm.2502009)
Supplement: Supplementary file 3 [file jm-2502009-Supplementary-Table-S3.pdf]

Table S3. Genome annotation of *Vibrio* phage vB_VhaS-MS03

Start	Stop	Strand	Predicted protein name	Closely related species	Query Coverage	E-value	Percent Identity	Accession No.
1	1974	+	Terminase large subunit	Vibrio phage vB_VcaS_HC	1.00	0.0E+00	1.00	QCG76653.1
1991	2233	+	Hypothetical protein	Vibrio phage vB_VcaS_HC	1.00	5.0E-53	1.00	QCG76652.1
2349	2612	+	Hypothetical protein	Vibrio phage D4	1.00	4.0E-50	0.93	UHD87231.1
2654	2920	+	Hypothetical protein	Vibrio phage vB_VcaS_HC	1.00	4.0E-55	0.95	QCG76650.1
2910	3323	+	Coil containing protein	Vibrio phage D4	0.91	6.0E-76	0.93	UHD87233.1
3351	5426	+	Pyruvate phosphate dikinase	Vibrio phage D4	1.00	0.0E+00	0.97	UHD87234.1
5431	5919	+	Hypothetical protein	Vibrio phage vB_VcaS_HC	1.00	8.0E-111	0.99	QCG76647.1
5912	6457	+	Putative protein-tyrosine phosphatase	Vibrio phage vB_VcaS_HC	1.00	5.0E-131	0.99	QCG76646.1
6483	7526	+	Hypothetical protein	Vibrio phage vB_VcaS_HC	1.00	0.0E+00	0.94	QCG76759.1
7537	8379	+	Transporter	Vibrio phage vB_VcaS_HC	1.00	0.0E+00	0.99	QCG76758.1
8379	10736	+	DNA polymerase I	Vibrio phage vB_VcaS_HC	1.00	0.0E+00	1.00	QCG76757.1
10738	12603	+	Portal protein	Vibrio phage vB_VhaS_R21Y	1.00	0.0E+00	1.00	WKV32866.1
12607	13695	+	Putative head morphogenesis protein	Vibrio phage vB_VhaS_R21Y	1.00	0.0E+00	1.00	WKV32867.1
13703	14353	+	DNA methyltransferase	Vibrio phage vB_VhaS_R21Y	1.00	1.0E-160	1.00	WKV32868.1
14537	14953	+	TMhelix containing protein	Vibrio phage vB_VcaS_HC	1.00	3.0E-97	1.00	QCG76753.1
14965	15420	+	Hypothetical protein	Vibrio phage vB_VhaS_R21Y	1.00	8.0E-107	1.00	WKV32870.1
15557	15952	+	Putative DNA polymerase I	Vibrio phage vB_VhaS_R21Y	1.00	3.0E-93	1.00	WKV32871.1
15955	16140	+	Hypothetical protein	Vibrio phage vB_VhaS_R21Y	1.00	6.0E-38	1.00	WKV32872.1
16121	16531	+	Hypothetical protein	Vibrio phage vB_VhaS_R21Y	1.00	7.0E-95	1.00	WKV32873.1
16542	16802	+	Hypothetical protein	Vibrio phage vB_VcaS_HC	1.00	1.0E-56	1.00	QCG76748.1
16809	17297	+	Hypothetical protein	Vibrio phage vB_VhaS_R21Y	1.00	3.0E-116	0.99	WKV32875.1
17300	17584	+	Hypothetical protein	Vibrio phage vB_VcaS_HC	1.00	5.0E-61	0.99	QCG76746.1
17590	17889	+	Hypothetical protein	Vibrio phage D4	1.00	7.0E-65	0.99	UHD87252.1

17974	18282	+	Hypothetical protein	Vibrio phage D4	1.00	2.0E-68	1.00	UHD87253.1
18334	19671	+	Hypothetical protein	Vibrio phage vB_VcaS_HC	1.00	0.0E+00	1.00	QCG76743.1
19717	20310	+	Hypothetical protein	Vibrio phage vB_VcaS_HC	1.00	7.0E-141	1.00	QCG76742.1
20321	20704	+	Hypothetical protein	Vibrio phage vB_VhaS_R21Y	1.00	5.0E-84	0.98	WKV32882.1
20714	20884	+	Hypothetical protein	Vibrio phage vB_VhaS_R21Y	1.00	4.0E-31	1.00	WKV32883.1
20881	21345	+	Putative zinc- or iron-chelating domain containing protein	Vibrio phage vB_VcaS_HC	1.00	3.0E-111	1.00	QCG76740.1
21326	21847	+	Hypothetical protein	Vibrio phage vB_VcaS_HC	1.00	8.0E-120	0.96	QCG76739.1
21847	22098	+	Hypothetical protein	Vibrio phage vB_VhaS_R21Y	1.00	1.0E-53	1.00	WKV32886.1
22076	22282	+	Hypothetical protein	Vibrio phage vB_VcaS_HC	0.86	5.0E-33	1.00	QCG76737.1
22754	23194	+	Hypothetical protein	Vibrio phage vB_VhaS_R21Y	1.00	1.0E-101	0.99	WKV32762.1
23182	23364	+	Hypothetical protein	Vibrio phage vB_VhaS_R21Y	0.98	3.0E-33	0.98	WKV32763.1
23361	24074	+	Hypothetical protein	Vibrio phage vB_VhaS_R21Y	1.00	2.0E-171	0.97	WKV32764.1
24055	24867	+	Coil containing protein	Vibrio phage vB_VcaS_HC	1.00	0.0E+00	0.98	QCG76733.1
24873	25181	+	Hypothetical protein	Vibrio phage vB_VcaS_HC	1.00	5.0E-69	1.00	QCG76732.1
25310	25816	+	Hypothetical protein	Vibrio phage vB_VcaS_HC	0.99	1.0E-112	0.96	QCG76731.1
25880	26662	+	Hypothetical protein	Vibrio phage vB_VhaS_R21Y	1.00	0.0E+00	0.99	WKV32768.1
26646	26804	+	Hypothetical protein	Vibrio phage D4	1.00	2.0E-28	1.00	UHD87270.1
26912	27106	+	Hypothetical protein	Vibrio phage vB_VcaS_HC	1.00	2.0E-37	1.00	QCG76729.1
27121	27450	+	Hypothetical protein	Vibrio phage vB_VcaS_HC	1.00	1.0E-73	0.99	QCG76728.1
27503	27898	+	Hypothetical protein	Vibrio phage vB_VhaS_R21Y	1.00	2.0E-92	0.98	WKV32772.1
27975	28355	+	Hypothetical protein	Vibrio phage D4	1.00	8.0E-84	0.98	UHD87274.1
28407	28868	+	Hypothetical protein	Vibrio phage D4	1.00	5.0E-109	0.97	UHD87275.1
28865	29197	+	TMhelix containing protein	Vibrio phage vB_VhaS_R21Y	1.00	8.0E-76	0.99	WKV32776.1
29181	29696	+	Hypothetical protein	Vibrio phage D4	1.00	2.0E-122	1.00	UHD87277.1
29683	30090	+	Hypothetical protein	Vibrio phage vB_VcaS_HC	1.00	2.0E-93	1.00	QCG76722.1
30096	30266	+	Hypothetical protein	Vibrio phage vB_VhaS_R21Y	1.00	1.0E-30	0.96	WKV32779.1

30661	30891	+	Hypothetical protein	Vibrio phage vB_VcaS_HC	1.00	6.0E-31	0.75	QCG76721.1
30903	31109	+	Hypothetical protein	Vibrio phage D4	1.00	8.0E-36	0.90	UHD87281.1
31206	31538	+	Hypothetical protein	Vibrio phage D4	1.00	2.0E-72	0.99	UHD87282.1
31597	31932	+	Hypothetical protein	Vibrio phage vB_VhaS_R21Y	1.00	8.0E-71	0.98	WKV32783.1
31989	32435	+	Hypothetical protein	Vibrio phage D4	1.00	2.0E-102	0.99	UHD87284.1
32425	32787	+	Hypothetical protein	Vibrio phage D4	1.00	3.0E-79	0.99	UHD87285.1
32833	33336	+	Hypothetical protein	Vibrio phage vB_VcaS_HC	1.00	2.0E-117	0.96	QCG76716.1
33471	33629	+	No significant similarity found					
33671	33976	+	Coil containing protein	Vibrio phage D4	1.00	1.0E-67	1.00	UHD87289.1
33980	34216	+	Hypothetical protein	Vibrio phage vB_VhaS_R21Y	1.00	3.0E-51	1.00	WKV32790.1
34229	34429	+	Hypothetical protein	Vibrio phage D4	1.00	4.0E-39	1.00	UHD87291.1
34429	34572	+	Hypothetical protein	Vibrio phage D4	1.00	3.0E-25	1.00	UHD87292.1
34696	34941	+	Hypothetical protein	Vibrio phage D4	1.00	1.0E-51	1.00	UHD87293.1
34957	35190	+	Hypothetical protein	Vibrio phage D4	1.00	3.0E-50	1.00	UHD87294.1
35317	35517	+	Hypothetical protein	Vibrio phage D4	1.00	1.0E-37	1.00	UHD87295.1
35564	35842	+	Hypothetical protein	Vibrio phage D4	1.00	4.0E-61	1.00	UHD87296.1
35839	36096	+	Hypothetical protein	Vibrio phage D4	1.00	5.0E-51	1.00	UHD87297.1
36093	37508	+	Hypothetical protein	Vibrio phage D4	1.00	0.0E+00	0.99	UHD87298.1
37602	38498	+	Hypothetical protein	Vibrio phage D4	1.00	0.0E+00	0.99	UHD87299.1
38573	39532	+	Hypothetical protein	Vibrio phage vB_VcaS_HC	1.00	0.0E+00	1.00	QCG76707.1
39663	39977	+	Hypothetical protein	Vibrio phage D4	1.00	2.0E-70	1.00	UHD87301.1
39955	40539	+	Coil containing protein	Vibrio phage D4	1.00	3.0E-140	1.00	UHD87302.1
40615	40977	+	Hypothetical protein	Vibrio phage D4	1.00	2.0E-81	1.00	UHD87303.1
41028	41420	+	Hypothetical protein	Vibrio phage D4	1.00	1.0E-87	1.00	UHD87304.1
41417	41851	+	Hypothetical protein	Vibrio phage D4	1.00	4.0E-103	0.99	UHD87305.1
41928	42281	+	Hypothetical protein	Vibrio phage D4	1.00	3.0E-78	1.00	UHD87306.1

42287	42781	+	Hypothetical protein	Vibrio phage D4	1.00	6.0E-118	0.99	UHD87307.1
42847	43542	+	Hypothetical protein	Vibrio phage vB_VcaS_HC	1.00	5.0E-168	1.00	QCG76699.1
43546	43827	+	Hypothetical protein	Vibrio phage vB_VcaS_HC	1.00	2.0E-61	1.00	QCG76698.1
43973	44263	+	Hypothetical protein	Vibrio phage vB_VcaS_HC	1.00	4.0E-63	1.00	QCG76697.1
44277	44579	+	Hypothetical protein	Vibrio phage vB_VcaS_HC	1.00	3.0E-69	1.00	QCG76696.1
44591	45115	+	Hypothetical protein	Vibrio phage vB_VcaS_HC	1.00	4.0E-127	1.00	QCG76695.1
45103	45387	+	Hypothetical protein	Vibrio phage vB_VcaS_HC	0.95	1.0E-58	0.99	QCG76694.1
45456	46244	+	Coil containing protein	Vibrio phage vB_VcaS_HC	1.00	0.0E+00	1.00	QCG76693.1
46237	48207	+	Hypothetical protein	Vibrio phage vB_VhaS_R21Y	1.00	0.0E+00	1.00	WKV32815.1
48273	48623	+	Hypothetical protein	Vibrio phage vB_VcaS_HC	1.00	2.0E-75	1.00	QCG76692.1
48752	49363	+	XkdF	Vibrio phage vB_VcaS_HC	1.00	5.0E-147	1.00	QCG76691.1
49366	50526	+	K+-dependent Na+/Ca+ exchanger	Vibrio phage vB_VhaS_R21Y	1.00	0.0E+00	1.00	WKV32818.1
50607	51557	+	Major capsid protein	Vibrio phage vB_VcaS_HC	1.00	0.0E+00	1.00	QCG76689.1
51635	51904	+	Coil containing protein	Vibrio phage vB_VcaS_HC	1.00	5.0E-58	1.00	QCG76688.1
51920	52564	+	Head completion adaptor	Vibrio phage vB_VcaS_HC	1.00	6.0E-154	1.00	QCG76687.1
52561	53025	+	Neck protein	Vibrio phage vB_VcaS_HC	1.00	3.0E-104	1.00	QCG76686.1
53022	53504	+	Tail-completion protein	Vibrio phage vB_VcaS_HC	1.00	5.0E-115	1.00	QCG76685.1
53558	54352	+	Major tail protein	Vibrio phage vB_VcaS_HC	1.00	0.0E+00	1.00	QCG76684.1
54441	54875	+	Hypothetical protein	Vibrio phage vB_VcaS_HC	1.00	3.0E-96	1.00	QCG76683.1
54962	55075	+	Hypothetical protein	Vibrio phage D4	1.00	8.0E-16	1.00	UHD87326.1
55081	59214	+	Tail length tape measure protein	Vibrio phage vB_VhaS_R21Y	1.00	0.0E+00	1.00	WKV32826.1
59211	59597	+	Hypothetical protein	Vibrio phage vB_VcaS_HC	1.00	1.0E-86	1.00	QCG76681.1
59607	60596	+	Hypothetical protein	Vibrio phage vB_VhaS_R21Y	1.00	0.0E+00	1.00	WKV32828.1
60606	61499	+	Hypothetical protein	Vibrio phage vB_VhaS_R21Y	1.00	0.0E+00	1.00	WKV32829.1
61502	62800	+	Hypothetical protein	Vibrio phage vB_VhaS_R21Y	1.00	0.0E+00	1.00	WKV32830.1
62803	63327	+	Hypothetical protein	Vibrio phage D4	1.00	3.0E-121	1.00	UHD87332.1

63328	63630	+	Hypothetical protein	Vibrio phage vB_VcaS_HC	1.00	7.0E-65	1.00	QCG76676.1
63627	63854	+	Hypothetical protein	Vibrio phage vB_VcaS_HC	1.00	8.0E-44	0.99	QCG76675.1
63844	64278	+	Hypothetical protein	Vibrio phage vB_VhaS_R21Y	1.00	5.0E-103	1.00	WKV32834.1
64280	64819	+	TMhelix containing protein	Vibrio phage vB_VcaS_HC	1.00	2.0E-125	1.00	QCG76673.1
64943	66166	+	AAA family ATPase	Vibrio phage vB_VcaS_HC	1.00	0.0E+00	1.00	QCG76672.1
66553	68040	+	Winged helix-turn-helix DNA-binding domain protein	Vibrio phage vB_VhaS_R21Y	1.00	0.0E+00	1.00	WKV32837.1
68040	68633	+	Hypothetical protein	Vibrio phage vB_VcaS_HC	1.00	2.0E-145	1.00	QCG76670.1
68618	70066	+	Replicative DNA helicase	Vibrio phage vB_VhaS_R21Y	1.00	0.0E+00	1.00	WKV32839.1
70138	70722	+	Hypothetical protein	Vibrio phage vB_VhaS_R21Y	1.00	2.0E-130	1.00	WKV32840.1
70715	71701	+	DNA primase	Vibrio phage vB_VhaS_R21Y	1.00	0.0E+00	0.99	WKV32841.1
71715	72632	+	Hypothetical protein	Vibrio phage vB_VhaS_R21Y	1.00	0.0E+00	1.00	WKV32842.1
72701	74107	+	Putative DNA helicase	Vibrio phage vB_VhaS_R21Y	1.00	0.0E+00	1.00	WKV32843.1
74206	74658	+	Putative DNA-binding domain protein	Vibrio phage vB_VhaS_R21Y	1.00	3.0E-105	1.00	WKV32844.1
74665	75048	+	Hypothetical protein	Vibrio phage vB_VcaS_HC	1.00	5.0E-90	1.00	QCG76663.1
75059	76135	+	RecA protein	Vibrio phage D4	1.00	0.0E+00	1.00	UHD87220.1
76116	76529	+	Hypothetical protein	Vibrio phage D4	1.00	3.0E-95	1.00	UHD87221.1
76519	77514	+	Rubredoxin-type fold protein	Vibrio phage vB_VhaS_R21Y	1.00	0.0E+00	1.00	WKV32848.1
77514	78068	+	Ribonuclease H-like domain protein	Vibrio phage D4	1.00	6.0E-134	1.00	UHD87223.1
78177	79151	+	Hypothetical protein	Vibrio phage vB_VcaS_HC	1.00	0.0E+00	1.00	QCG76658.1
79218	79487	+	Hypothetical protein	Vibrio phage vB_VcaS_HC	1.00	3.0E-57	1.00	QCG76657.1
79512	80255	+	Hypothetical protein	Vibrio phage vB_VcaS_HC	1.00	0.0E+00	1.00	QCG76656.1
80333	81064	+	Hypothetical protein	Vibrio phage vB_VcaS_HC	1.00	0.0E+00	1.00	QCG76655.1
81200	81045	-	No significant similarity found					
